# Investigating the potential effects of α-synuclein aggregation on susceptibility to chronic stress in a mouse Parkinson’s disease model

**DOI:** 10.1007/s43440-023-00530-z

**Published:** 2023-09-19

**Authors:** Anna Alwani, Katarzyna Maziarz, Gabriela Burda, Monika Jankowska-Kiełtyka, Adam Roman, Gabriela Łyszczarz, Safak Er, Justyna Barut, Olga Barczyk-Woźnicka, Elżbieta Pyza, Grzegorz Kreiner, Irena Nalepa, Piotr Chmielarz

**Affiliations:** 1 Department of Brain Biochemistry, Maj Institute of Pharmacology, Polish Academy of Sciences, Smętna 12, 31-343 Kraków, Poland; 2 Faculty of Pharmacy, Drug Research Program, University of Helsinki, Helsinki, Finland; 3 Institute of Biotechnology, HiLIFE, University of Helsinki, Helsinki, Finland; 4 Department of Cell Biology and Imaging, Institute of Zoology and Biomedical Research, Jagiellonian University, Gronostajowa 9, 30-387 Kraków, Poland

**Keywords:** α-synuclein, Preformed fibrils, Parkinson’s disease, Non-motor symptoms, Depression, Chronic stress

## Abstract

**Background:**

Parkinson’s disease (PD) is a motor disorder characterized by the degeneration of dopaminergic neurons, putatively due to the accumulation of α-synuclein (α-syn) in Lewy bodies (LBs) in Substantia Nigra. PD is also associated with the formation of LBs in brain areas responsible for emotional and cognitive regulation such as the amygdala and prefrontal cortex, and concurrent depression prevalence in PD patients. The exact link between dopaminergic cell loss, α-syn aggregation, depression, and stress, a major depression risk factor, is unclear. Therefore, we aimed to explore the interplay between sensitivity to chronic stress and α-syn aggregation.

**Methods:**

Bilateral injections of α-syn preformed fibrils (PFFs) into the striatum of C57Bl/6 J mice were used to induce α-syn aggregation. Three months after injections, animals were exposed to chronic social defeat stress.

**Results:**

α-syn aggregation did not affect stress susceptibility but independently caused increased locomotor activity in the open field test, reduced anxiety in the light–dark box test, and increased active time in the tail suspension test. Ex vivo analysis revealed modest dopaminergic neuron loss in the substantia nigra and reduced dopaminergic innervation in the dorsal striatum in PFFs injected groups. α-Syn aggregates were prominent in the amygdala, prefrontal cortex, and substantia nigra, with minimal α-syn aggregation in the raphe nuclei and locus coeruleus.

**Conclusions:**

Progressive bilateral α-syn aggregation might lead to compensatory activity increase and alterations in emotionally regulated behavior, without affecting stress susceptibility. Understanding how α-syn aggregation and degeneration in specific brain structures contribute to depression and anxiety in PD patients requires further investigation.

**Supplementary Information:**

The online version contains supplementary material available at 10.1007/s43440-023-00530-z.

## Introduction

Parkinson’s disease (PD) is the most common, spontaneous, and slowly progressing motor disorder, predominantly affecting the elderly population. It is characterized by the presence of motor symptoms, such as bradykinesia or akinesis, rigidity of muscles, postural instability, and progressing cognitive impairment [1, 2]. Motor symptoms in PD are largely attributed to the degeneration of dopaminergic (DA) neurons in the nigrostriatal pathway, especially in the pars compacta of the substantia nigra (pc-SN; SN). This degeneration is linked with the accumulation of pathological, misfolded α-synuclein (α-syn) in Lewy Bodies (LBs) within the cytoplasm of neurons and in neuritic inclusions called Lewy neurites (LNs) [2, 3] collectively termed Lewy pathology. Other factors, genetic and environmental or specific physiology of DA neurons might also significantly contribute to their demise [4].

Currently, PD progression is also associated with the development of psychological disturbances such as sleep disorder, anxiety, apathy, cognitive impairments, or depression [1, 5]. These coincide with the spread of Lewy pathology into areas of the central nervous system responsible for the regulation of cognition and emotional processes, such as the prefrontal cortex and amygdala nuclei [3, 6–8].

Among non-motor symptoms of PD, depression is one that significantly contributes to the disease burden. Approximately, 35–50% of PD patients are diagnosed with depression and there is also a significant increase in anxiety disorders among this population [9–11]. Similar increases in depression incidence are also observed in another synucleinopathy—Dementia with Lewy bodies (DLB) [12].

Depression is often prodromal to the clinical diagnosis of PD, preceding motor symptoms [11]. Yet, it is widely accepted that pathological processes in the brains of PD patients start many years before clinical diagnosis [1, 13]. By the time the motor symptoms mainfest, Lewy pathology is already widespread in the brain, while a large number of DA neurons are lost along with their projections to the SN.

Depression has been traditionally linked with deficits in monoaminergic signaling [14]—which are also present in PD patients. However, there have been alternative hypotheses of depression more focused on neuroplasticity changes in limbic structures like the amygdala and prefrontal cortex [14]. The prefrontal cortex and amygdala have also been reported to be strongly affected in PD, albeit there have been some conflicting results on the subject [3, 15–17]. It has also been proposed that different subtypes of PD might be characterized by distinct patterns of Lewy pathology spread [18] perhaps explaining conflicting results of other studies and variable incidence of non-motor symptoms.

Recently, Lewy pathology in PD has been modeled by injection of exogenously formed, prion-like α-syn preformed fibrils (PFFs) [19]. However, there has been little focus on depression and anxiety symptoms in these studies. Most were conducted using unilaterally injected animals [20], while clinical data suggest that there is a higher prevalence of depression when pathology is bilateral, which happens presumably when it starts in the periphery [18, 21].

In non-PD linked depression, chronic stress is a major risk factor [22, 23]. Chronic stress paradigms and injections of stress hormones are common methods of modeling depression in animals. Surprisingly, little is known about the possible interaction of stress, PD, and Lewy pathology.

**Fig. 1:**
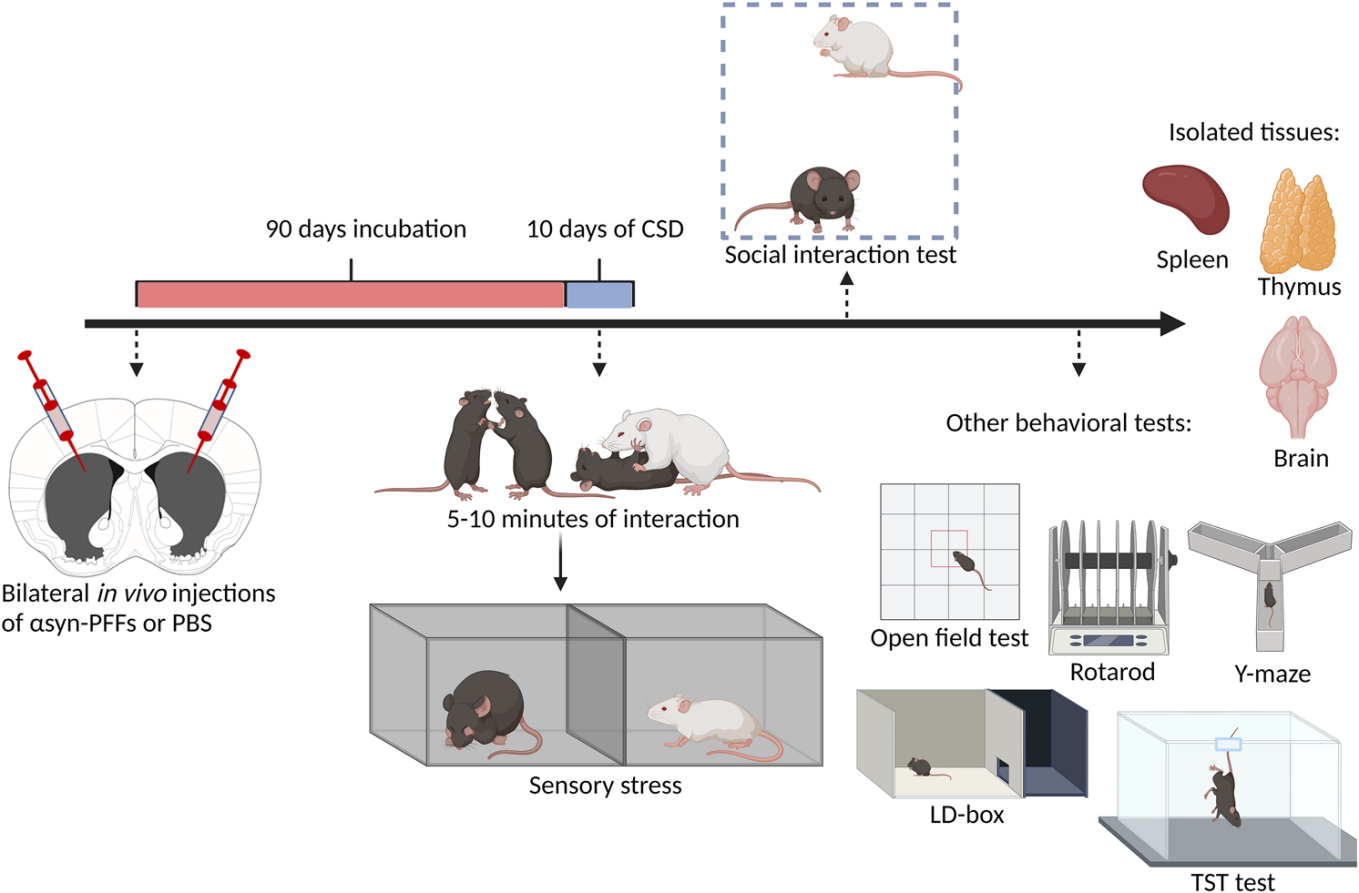
Timeline of the experiments. Animals were bilaterally injected with mouse WT αsyn-PFFs into the striatum, followed by 90 day incubation period. Afterward, a 10-day chronic social defeat (CSD) stress procedure was applied following a social interaction test to assess susceptibility to stress. This was followed by a panel of behavioral tests: Open Field test, Rotarod, Light–dark box test, Y-maze spontaneous alternation test, and Tail Suspension test. After the behavioral experiments, animals were sacrificed and the brain, thymus, and spleen were collected for ex vivo analyses

Here, we explore if there is an interaction between the presence of α-syn inclusions in the mice brains and their sensitivity to chronic social stress. We hypothesized that α-syn pathology in form of Lewy Body like aggregates, especially in limbic areas, affects the susceptibility to stress in mice model of α-synucleinopathy. We have modeled the propagation of Lewy pathology by bilateral injections of α-syn-PFFs, followed by 3 months of incubation period—time after which significant motor dysfunctions are not yet observed; however, Lewy pathology is already widespread [24]. At this time point, which could be related to a pre-symptomatic stage of PD, we subjected the animals to a chronic social defeat stress paradigm and a panel of behavioral tests (Fig. 1). Thus, we tested if the presence of Lewy pathology might affect susceptibility to stress. Moreover, we have also investigated the inverse possibility that chronic stress might exacerbate aggregation of α-syn or loss of dopamine neurons.

## Materials and methods

### Preparation and validation of* α-syn fibrils*

Recombinant mouse WT α-syn monomers were obtained from Proteos (#RP-009). Monomer at 5 mg/mL was aggregated under shaking for 7 days at 37 °C according to the described protocol [25]. The obtained fibrils were diluted in 1 × PBS to a final concentration of 2 mg/mL and sonicated with a high-power probe sonicator (UP100H, Hielscher) with the following settings: 2 mm sonicator probe, 60 s with maximum power pulses, and 0.5 s on/0.5 s off on ice. The size of obtained fibrils was confirmed to be ~ 50 nm in length by transmission electron microscopy (Jeol JEM2100), and their functionality to induce aggregation of endogenous α-syn was tested on primary dopaminergic cultures as described before [26].

### Animals and housing

The experiments were performed on adult C57BL/6 J male mice (12 weeks old). Additionally, male retired breeder CD-1 mice were used in chronic social defeat stress procedure and social interaction tests. All animals were housed at standard conditions at room temperature 22 ± 2 °C, 55 ± 10% humidity, and 12 h light/dark cycle, with food and water ad libitum. All animal experiments were approved by the Local Ethical Commission for Animal Experiments at Maj Institute of Pharmacology, Polish Academy of Sciences in Krakow (permit number 52/2019) and fulfilled the requirements of the EU Directive 2010/63/EU on the protection of animals used for scientific purposes.

### Stereotaxic injections

Stereotaxic surgeries were performed under ketamine (80 mg/kg)/xylasine (10 mg/kg) anesthesia. Animals were injected with α-syn-PFFs (2 mg/mL) bilaterally at coordinates A/P 0.7 mm, M/L 2.2 mm and − 2.2 mm, D/V − 3.0, 2.5 µL at 0.2 µL/min; after the injection, the needle was allowed to sit in place for another 3 min.

### Behavioral procedures

Male C57Bl6/J mice were randomly divided into two groups, and injected stereotaxically into the striatum with either PBS or αsyn-PFFs. Subsequently, both PBS and αsyn-PFFs injected mice were further subdivided into stress and non-stressed groups and subjected to treatment according to the protocol described below. After 10 days of the chronic social defeat procedure, the Social Interaction (SI) test was used to assess the effects of stress, followed by a panel of behavioral tests, tissue collection, and ex vivo analyses (see below).

#### The chronic social defeat stress procedure

C57Bl/6 J mice were placed in a housing cage separated in half by clear plexiglass preventing the mouse from going to the other side of the cage but with the holes allowing sensory stimuli to pass through. The cage contained 1 mouse on each of the separated sides, each with access to water and food ad libitum. For non-stressed groups, C57Bl/6 J control mice were placed in both cage compartments for 10 days. The stressed groups had a C57Bl/6 J mouse on one side and a selected aggressive male CD-1 on the other. Each day, for 10 min a day, a C57Bl/6 J mouse was placed in the compartment along with a CD-1 mouse. Mouse contact was monitored and terminated in case there was a risk of injury from mice fighting. Each day, for 10 consecutive days, the C57Bl/6 J mouse was moved to a different cage with a different CD-1 mouse.

#### Social interaction test

C57Bl/6 J mouse was placed in the square 40 × 40 cm arena, with a small enclosure placed inside touching one of the walls in the middle. The enclosure was made from wire mesh allowing contact between the mice, within the enclosure. Interactions of C57Bl/6 J mice with the empty enclosure and with the enclosure with CD-1 mice placed inside was scored in two 2.5-min consecutive test. CD-1 mice placed inside the enclosure were always different than any of the CD-1 mice who were previously housed as aggressor mice with given C57Bl/6 J during chronic social defeat procedure. Active exploring of the enclosure by the C57Bl/6 J mouse was scored by the experimenter, and the ratio of time spent exploring the enclosure with the CD1 mouse/empty enclosure was calculated. A ratio lower than 1 indicated avoidance of the contact with other mice, which is one of the outcomes of the chronic social defeat stress procedure.

#### Open field test

Animals were video recorded for 15 min in 40 × 40 cm square boxes, under 300 lx illumination, and the total distance moved was quantified.

#### Rotarod test

Motor coordination was tested on the accelerated rotarod (Ugo Basile). Animals were pretrained by placing them on a shaft rotating at a constant speed (4 rpm) for 5 min, 24 h before the actual test. In the test procedure, the rotating shaft automatically accelerated in the range of 4–40 rpm in 300 s. The time when the mouse fell from the shaft was recorded.

#### Tail suspension test

To assess depressive-like behavior; the time when the animals were active or immobile while suspended by the tail for 6 min was measured. Scoring of the test was done automatically with Noldues EthoVision XT8 as described previously [27]

#### Light–dark box test

Anxiety was measured by placing the mouse for 5 min in a box divided into two compartments—bright, illuminated about 300 lx, and dark (about 20–30 lx). The time spent in the bright zone was measured.

#### Y-maze spontaneous alternation test

To assess working memory, mice were placed in a Y-maze for 5 min. Spontaneous exploration of the successive arms is observed and the mouse’s tendency to visit the previous arm relative to the next arm is scored (spontaneous alternations). The ratio of spontaneous alternations, to total possible alternations, was calculated as the index of working memory function.

### Tissue processing and staining

Animals were sacrificed, and their brains were extracted and fixed in 4% paraformaldehyde (PFA) for 48 h, and stored in 0.4% PFA. The brains were cut on a vibratome (Leica, Germany) into 50 µm sections, collected into PBS on multiwell plates, and stored at 4 °C. To assess the number of dopamine neurons every 4^th^ section from the entire midbrain, encompassing SN/VTA areas were incubated with anti-tyrosine hydroxylase (TH) (1:2000, AB1542, Millipore,) overnight at 4 °C. This was followed by incubation with biotinylated secondary antibody and visualized with the Avidin/Biotin Complex (ABC; Vector Laboratories, USA) and diaminobenzidine staining (DAB; Sigma, USA). The sections were imaged a Nikon Eclipse 50i microscope. For assessment of the burden of aggregated α-syn selected sections from the corresponding bregma area spanning the frontal cortex, amygdala, and SN/VTA were incubated with anti-pSer129α-syn (1:4000, AB51253, Abcam) overnight at 4 °C. This was followed by incubation with biotinylated secondary antibody and visualized with the Avidin/Biotin Complex (ABC; Vector Laboratories, USA) and diaminobenzidine staining (DAB; Sigma, USA) and counterstained with Methyl Green (FD Neurotech) accordingly to the manufacturer’s instruction. Sections were imaged on a histological slide scanner (Apeiro) and quantified as described below. For assessment of α-syn pathology burden in noradrenergic and serotonergic areas, chosen corresponding sections from LC and RN were stained with rabbit anti-pSer129α-syn (1:2000, AB51253, Abcam) and anti-tyrosine hydroxylase (TH) (1:2000, Millipore, AB1542) or anti-Tryptophan Hydroxylase (TpH) (1:1000, AB1541 Millipore) and proper secondary fluorescent antibodies. While TH is also expressed in other cell types, i.e., DA neurons, the analyzed sections were restricted to the area of LC where only noradrenergic neurons express TH. The sections were mounted on mounted with Vectashield (Vector, H-1500) and imaged in wide field mode on Leica TCS SP8 and quantified in Fiji/ImageJ [28].

### Image analysis

Images of mouse brain slices were obtained by Leica Apeiro Slide Scanner and images from corresponding areas were selected accordingly to The Mouse Brain in Stereotaxic Coordinates (Paxinos and Franklin 2001, 2nd Edition). In the case of TH, DAB IHC staining in the dorsal and ventral striatum (analyzed area: Bregma ~ 0.86), images were converted to an 8-bit gray scale to measure the Mean Gray Value parameter of the selected areas (via freehand selection and ROI manager). Evaluation of PFF aggregation level in the amygdala (analyzed area: Bregma = from − 1.06 to − 2.06), cortex [Prelimbic (PL) and Infralimbic (IL) area] (analyzed area: Bregma = from 1.54 to − 1.70), and pc-SN (analyzed area: Bregma = from − 2.92 to − 3.08) was carried out using Trainable Weka Segmentation 2D (TWS) plugin [29] in FIJI enabling to separate the LBs, LNs, Cells (without PFF pathology), and background. Next, TWS-generated images were converted to an 8-bit gray scale, thresholded, and converted to mask to measure the count and Area of LBs and Area of LNs and LBs + LNs of the selected areas (via freehand selection and ROI manager (Figure S1). The images were processed using Fill Holes and Watershed options in case of count and Area of LBs measurements. Quantification αsyn in serotonergic neurons was done manually in FIJI/ImageJ. TpH or TpH and pS129-αsyn positive cells were identified and quantified. Then, the percentage of pS129-αsyn-positive cells in this total count was calculated.

### Quantification of splenocytes and thymocytes

After animals were sacrificed the abdominal side of the body was disinfected with 70% ethyl alcohol (POCh, Poland). The abdomen was dissected with sterile instruments, and the spleen and thymus were dissected. Organs were weighed under sterile conditions, then transferred to a dish with cold PBS, and kept on ice. The relative weights of the organs were calculated per mg of the body weight. As a measure of the overall activity of the immune system, the number of thymocytes and splenocytes per unit mass of the thymus and spleen, respectively, was calculated and expressed in thousands of cells contained in 1 mg of the tissue. The viability of thymocytes was quantified by Propidium Iodine uptake assay on an FACSCanto II flow cytometer using FACSDiva software (both Becton Dickinson, San Jose, USA) as described previously [30].

### Statistical analysis

Data from behavioral experiments, the number of TH + cells in the SN and the VTA, analysis of the spleen and the thymus parameters, and intensity differences of the striatal TH staining between control and α-syn-PFFs and non-stressed and stressed groups were determined by two-way ANOVA, followed by LSD multiple comparisons post hoc test. The evaluation of LB and LN inclusions after αsyn-PFF striatal injections between non-stressed and stressed groups was determined by the Student’s unpaired two-tailed t test. Outliers were detected with the Grubbs test. All statistics were done in GraphPad Prism software (version 10.0.0).

## Results

### Injection of αsyn-PFFs increases locomotor activity and decreases anxiety-like behavior without interaction with chronic stress

**Fig. 2:**
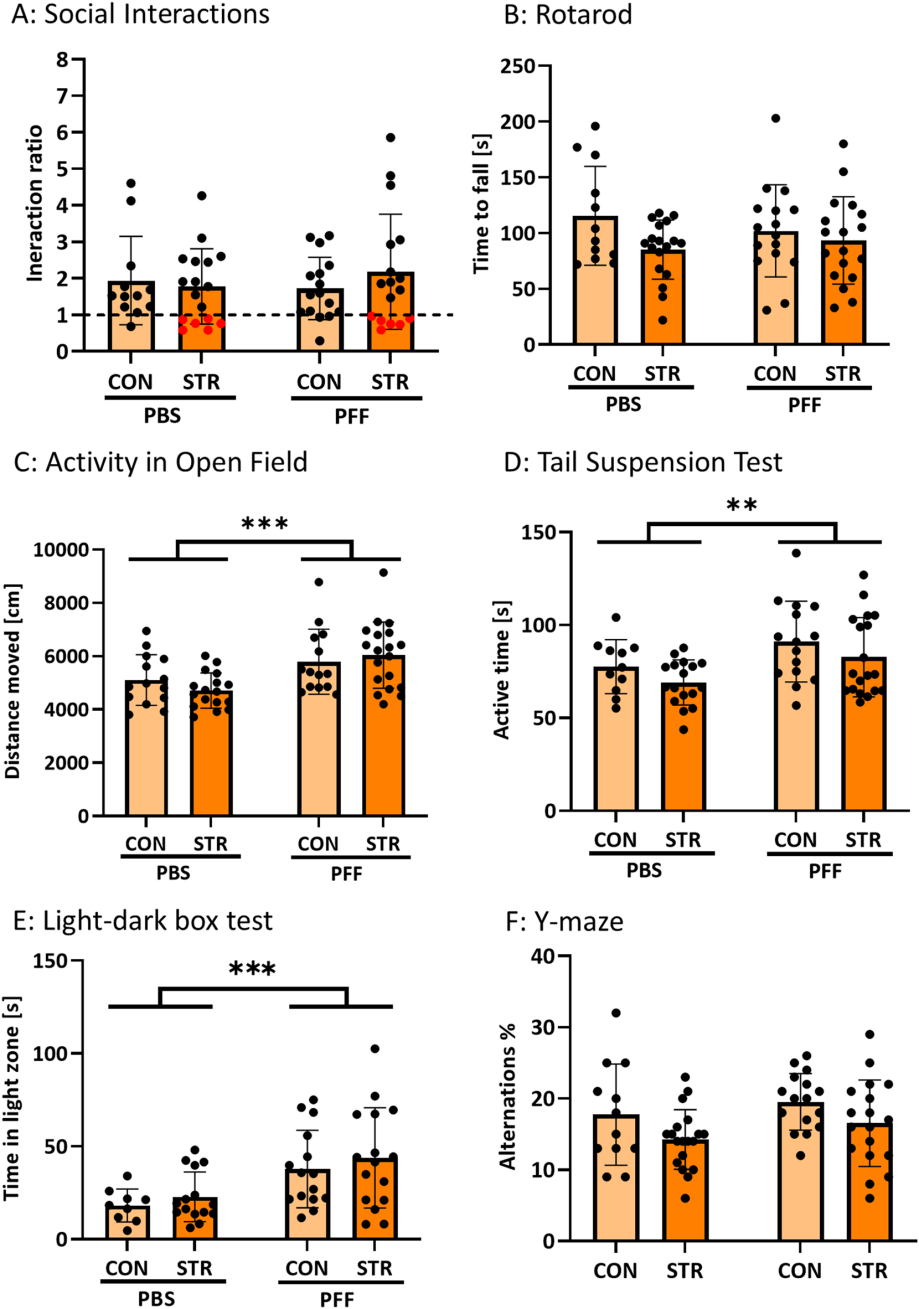
Behavioral characterization of mice injected with αsyn PFFs and subjected to chronic social defeat stress. α-Syn PFFs (PFF) and PBS-injected animals were divided either into control (CON) or subjected to chronic social defeat stress (STR). **A** PBS and PFF-injected animals did not differ in the Social Interaction test. Stressed animals of both PBS and PFF groups responded similarly with six animals from each group exhibiting decreased social interactions (as indicated by an interaction index lower than 1, marked by red dots on graphs). **B** Rotarod test has not shown significant motor coordination deficits in any of the groups. **C** Traveled distance in Open Field test was higher in α-syn-PFFs injected animals, compared to PBS-injected animals. **D** αsyn-PFFs injected animals show highest time spent active in Tail Suspension Test (TST), compared to PBS-injected animals. **E** Similarly, αsyn-PFFs injected mice show lower anxiety-like behavior in the light–dark box (LD-box) test. **F** No effects of stress or αsyn-PFFs on working memory were observed in Y-maze spontaneous alternation test. *N* = 12–19. Data are mean ± SD, **p < 0.01, ***p < 0.001 for main effect of PFFs (two-way ANOVA)

Stress indeed elicited a decrease in social interaction index in a subset of animals as indicated (Fig. 2A, red dots); however, there were no differences between PBS and PFF groups, neither in control nor chronically stressed mice. Furthermore, mice in all groups demonstrated normal motor coordination in the Rotarod test (Fig. 2B). However, in the Open Field test (Fig. 2C, Fig. S2), αsyn-PFFs injected animals demonstrated a longer traveled distance [(*F*_1,60_ = 14,45, *p* = 0.0003) for αsyn-PFFs effect in 2-way ANOVA]. Similarly, αsyn-PFFs injected animals showed more time active in the TST test (Fig. 2D) (*F*_1,58_ = 8.166, *p* = 0.0059). The effect of stress in TST was modest, with slightly lower active time in both PBS-stress and αsyn-PFFs-stress groups as compared to respective controls; however, this effect did not reach statistical significance in 2-way ANOVA (*F*_1,58_ = 3.195, *p* = 0.0791). There was also a main effect of αsyn-PFFs (*F*_1,51_ = 13.57, *p* = 0.0006) but no effect of stress in the LD-box test (Fig. 2E). Finally, neither αsyn-PFFs nor stress significantly affected the working memory of mice tested in the Y-maze (Fig. 2F). Finally, 2-way ANOVA has not shown any interactions between chronic stress and αsyn-PFFs’ injections in any of the behavioral tests.

### αsyn-PFFs decrease the number of tyrosine hydroxylase-positive cells in substantia nigra but not ventral tegmental area, and decreased dopaminergic innervation in the dorsal striatum, independently of stress exposure

**Fig. 3:**
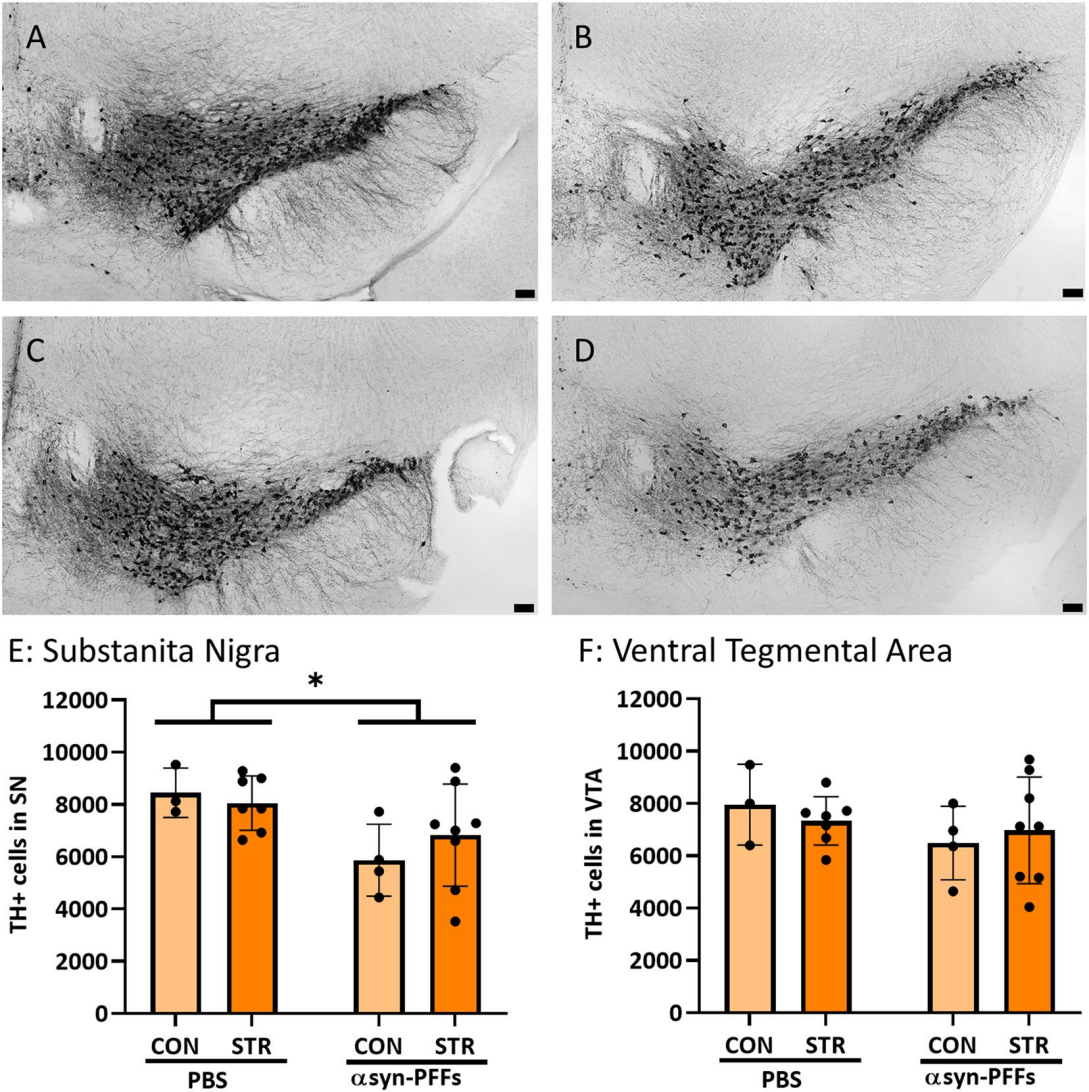
Immunohistochemical evaluation of Tyrosine Hydroxylase positive (TH +) neurons in the Substantia Nigra (SN) and the Ventral Tegmental Area (VTA) of unstressed control (CON) or chronically stressed (STR) animals which were injected bilaterally either with PBS or αsyn-PFFs (PFF). A–D) representative images of Tyrosine Hydroxylase stained brain sections from: **A** CON/PBS animals, **B** CON/PFF animals, **C** STR/PBS animals, and **D** STR/PFF animals. **E** Quantification of TH + cells in the SN shows decreased number of cells in αsyn-PFFs injected animals regardless of stress. **F** There was no significant decrease in TH + cells in the VTA. *N* = 3–8. Data are mean ± SD, *p < 0.05 (two-way ANOVA). Scale bars = 100 µm

Immunohistochemical evaluation of the brains dissected from experimental animals demonstrated a modest but significant decrease in the SN (*F*_1, 18_ = 7.569, *p* = 0.0131) but not in the Ventral Tegmental Area (VTA) (Fig. 3) of the numbers of cells immunopositive for Tyrosine Hydroxylase (TH +), an enzyme critical for the synthesis of dopamine.

**Fig. 4:**
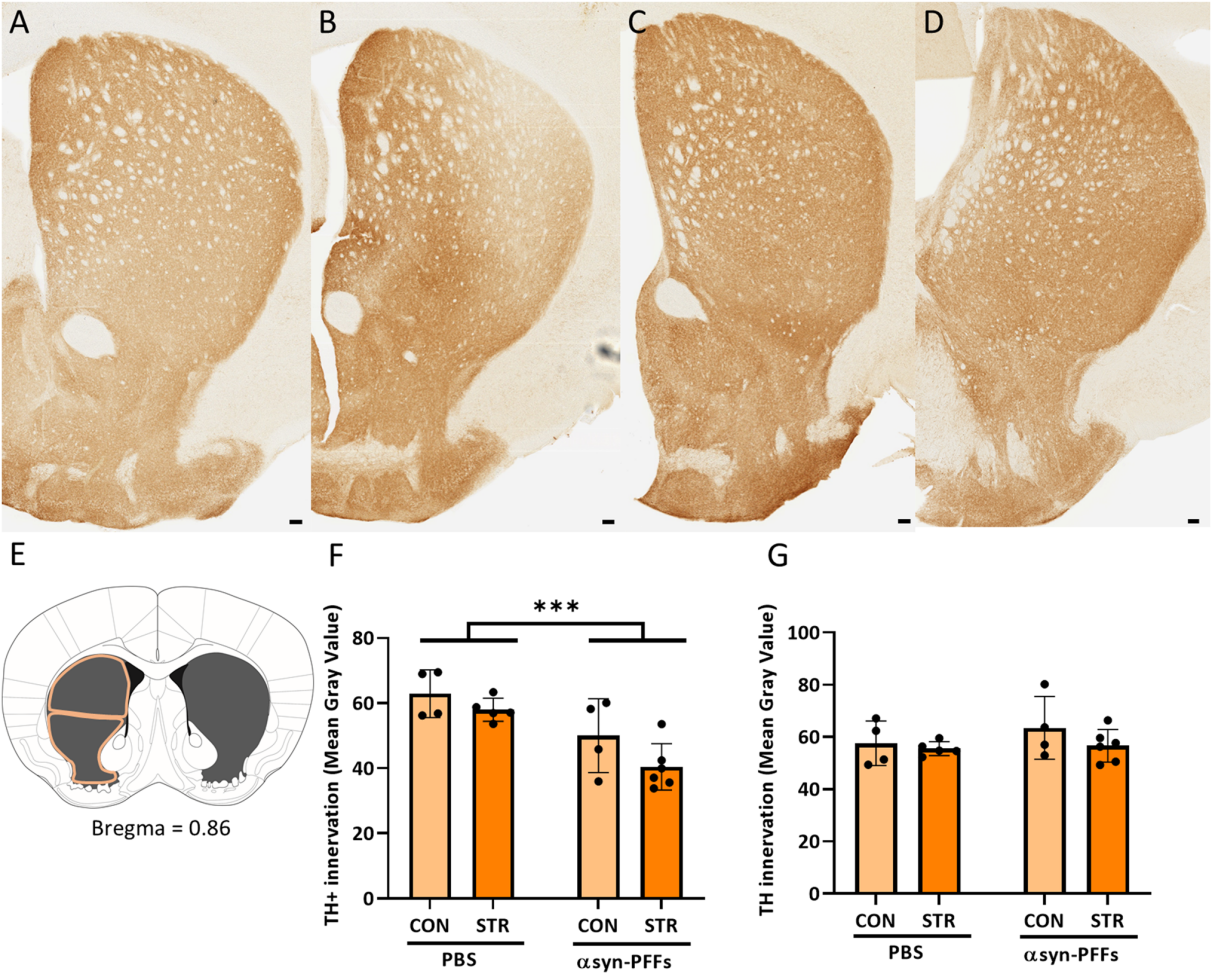
Immunohistochemical evaluation of Tyrosine Hydroxylase positive (TH +) fibers (brown) in the striatum of unstressed control (CON) or chronically stressed (STR) animals which were injected bilaterally either with PBS or αsyn-PFFs (PFF). Representative images of Tyrosine Hydroxylase stained brain sections from: **A** CON/PBS animals **B** STR/PBS animals, **C** CON/PFF animals, and **D** STR/PFF animals. **E** Schematic representation of the quantified area (in gray) with the dorsal and ventral striatum outlined. **F** Quantification of TH + fiber density in the dorsal striatum showed decreased innervation in αsyn-PFFs injected animals regardless of stress. **G** There was no significant decrease in TH + fiber density in the ventral striatum. *N* = 4–6. Data are mean ± SD, *p < 0.05 (two-way ANOVA). Scale bars = 100 µm

In agreement with the loss of TH + cells from the SN, we also observed decreased TH immunoreactivity after PFF treatment in the dorsal striatum (Fig. 4A–D, F) (*F*_1, 15_ = 18.81, *p* = 0.0006 for the main effect of αsyn-PFFs in 2-way ANOVA) which is the main projection site of dopaminergic neurons in the SN, while there was no difference between groups in the ventral striatum that is innervated mainly by projections from VTA neurons (Fig. 4A–D, G). There were no significant effects of chronic stress neither on TH+ cell number in the SN and VTA areas nor on fiber density in the striatum nor on the interaction between stress and αsyn-PFFs’ injection in these parameters.

### The evaluation of the αsyn-PFFs aggregation level in the amygdala, prelimbic (PL) and infralimbic (IL) cortex, and pars compacta of the substantia nigra (pc-SN) of αsyn-PFFs injected animals

Next, we evaluated the level of PFF-induced αsyn inclusions similar to LBs and LNs in the pc-SN (Fig. S3) and brain areas associated with the regulation of cognition and emotional processes (amygdala Fig. S4), and prelimbic (PL) and infralimbic (IL) cortices (Fig. S5). We have quantified the number of large circular inclusions resembling early stage LBs, the area covered by those inclusions, as well as the area covered by elongated inclusions resembling LNs. Our findings revealed no significant difference in the level of LB and LN inclusions in the investigated brain structures between stressed and non-stressed groups (Fig. S3–S5).

### Effects of αsyn-PFFs injections and exposure to stress on noradrenergic and serotonergic neurons

To verify the effect of αsyn-PFFs injections on the degeneration of DA neurons and αsyn-PFFs aggregation levels in the SN neurons, noradrenergic neurons of Locus Coeruleus (LC), and serotonergic neurons in the Raphe Nuclei (RN), immunofluorescent staining was performed for the colocalizing markers of these cells (TH and Tryptophan Hydroxylase, TpH, respectively) within the obtained mouse brain slices (Fig. S6). We did not observe any αsyn aggregates in the LC area (Figure S6A), while there was only a minimal amount of αsyn aggregates in the serotonergic neurons in the RN (Fig. S6B). There were no statistically significant differences in the number of TpH positive cells harboring αsyn aggregates (Fig. S6C) nor in the total number of TpH positive cells (Fig. S6D) between control and stress groups of αsyn-PFFs injected animals.

### Neither injection of αsyn-PFFs nor chronic stress adversely affected the peripheral immune system

**Fig. 5:**
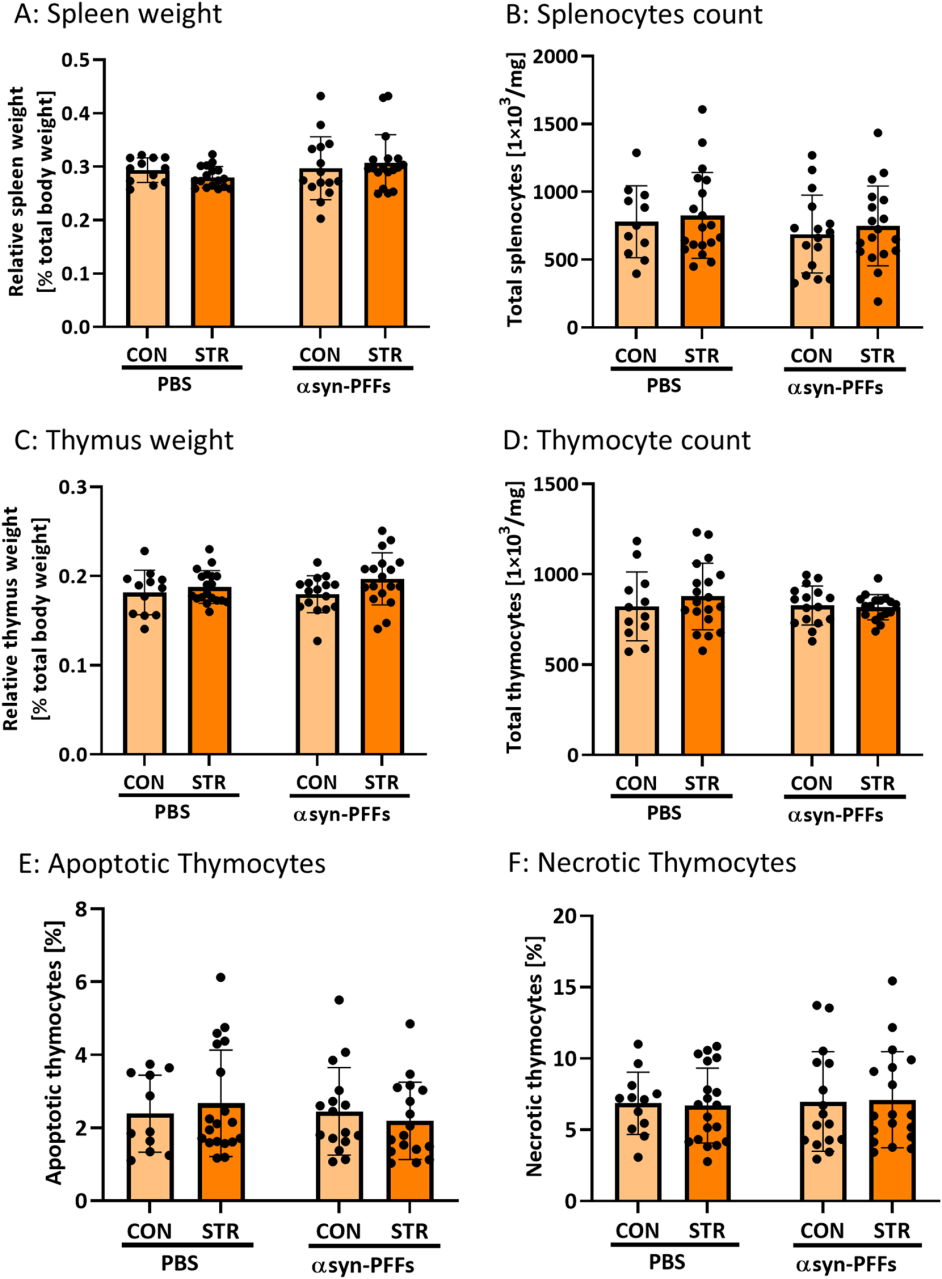
Assessment of spleen and thymus function parameters as markers of the peripheral immune system in mice injected with αsyn PFFs and subjected to chronic social defeat stress. α-syn-PFFs (PFF) and PBS-injected animals were divided either into control (CON) or subjected to chronic social defeat stress (STR). **A** spleen weight in relation to total body weight, **B** number of splenocytes per mg of the spleen tissue, **C** thymus weight in relation to total body weight, **D** number of thymocytes per mg of the thymus tissue, **E** number of apoptotic thymocytes, and **F** number of necrotic thymocytes were quantified. No significant effects of stress or PFFs were observed. *N* = 11–19. Data are mean ± SD

Intense chronic stress can affect the peripheral immune system and related organs like the spleen and thymus through the action of stress hormones. We investigated the effects of chronic social defeat stress and αsyn-PFF injections on the function of the spleen—by measuring the total spleen size (Fig. 5A) and the number of splenocytes (Fig. 5B), as well as on the function of the thymus by measuring relative thymus size (Fig. 5C), the number of thymocytes (Fig. 5D) and quantified the rates at which they undergo apoptotic (Fig. 5E) and necrotic cell death (Fig. 5F). Neither chronic social defeat stress nor αsyn-PFFs injections affected the measured parameters, indicating that the stress procedure itself was not severe enough to affect the immune system function neither in PBS nor in αsyn-PFF-injected animals.

## Discussion

Our data show that bilateral intrastriatal injections of α-syn-PFFs elicit a modest loss of dopamine cells and striatal dopaminergic innervation, and prominent Lewy pathology in the SN, amygdala, and throughout the cortex, together modeling to some extent a pre-motor PD image. This is linked with increased locomotor activity and unexpected alterations in anxiety and depressive-like behaviors, which might reflect some of the early compensatory changes either in the dopaminergic system or affected limbic areas. However, observed pathological changes fails to elicit increased susceptibility to chronic stress.

Increase in locomotor activity, an increase in active time in Tail Suspension Test (TST) and decreased anxiety in LD-box test were independent of the stress, thus elicited either by the observed, modest, loss of dopamine neurons, or dysfunctions of cells in other brain areas where Lewy pathology was present—namely the amygdala and prefrontal cortex. At first, it may seem surprising that loss of dopamine cells would increase locomotor activity; however, it is conceivable that the observed 20% loss of dopamine neurons and a similar loss of dopaminergic striatal innervation could lead to adaptive changes in either the remaining dopaminergic neurites or dopamine receptor and uptake systems; in fact, such changes are known to occur to compensate much larger cell loss [32]. Moreover, there have been studies demonstrating a compensatory increase in striatal dopamine release after moderate, but not severe, neurotoxin-induced dopaminergic neuron degeneration [33] which reached almost 3 times higher levels in toxin treated than in control animals. There have been also reports of a compensatory decrease in dopamine elimination rate [34]. Our study suggests that similar compensatory adaptation might be present in the early stages of Lewy Pathology development and prompts further investigations into changes in levels of dopamine synthesis enzymes, transporters, and receptors as well as functional studies measuring dopamine release in striatum at early time points after stages of α-syn-PFFs injections.

Longer activity time in TST and more time spent in the light compartment in the LD-box test could be interpreted as decreased depressive-like behavior and decreased anxiety, respectively, in PFF-injected animals as compared to PBS-injected groups. However, TST results should be interpreted cautiously since increased time active in TST might also reflect a general change in the activity, which was indicated by the Open Field Test. Nonetheless, our results are one of the first to show that the early stage of PFF-induced Lewy pathology with concomitant modest dopamine system degeneration can cause alterations in emotionally related behavior such as anxiety, suggesting that the pathology affects brain areas important in emotional control. While the direction of changes was surprising, it is actually in line with a recent study by Stoyka et al. [35] showing a decrease in fear conditioning-related freezing, which was interpreted as an impaired acquisition of the fear memory. One can also hypothesize that decreased anxiety might be explained by early compensatory changes. There have been relatively few other studies exploring depression and anxiety in preclinical models of PD with α-syn aggregation. Some studies revealed the cognitive and behavioral phenotypes in transgenic mice models of α-synucleinopathy, based on overexpression of α-syn, where difficulties were observed in rule-reversing tests, spontaneous alternation, and increased anxiety [36–38]. However, it should be noted that while there is an aggregation of α-syn in the transgenic overexpression models, these models do not recapitulate the spreading nature of the pathology, nor formation of proper LBs but rather demonstrate diffuse aggregation of α-syn [39].

Our data also show that neither Lewy pathology in the SN, prefrontal cortex, or amygdala, nor the modest loss of DA neurons is enough to increase susceptibility to chronic stress. This does not fully preclude that there might be increased susceptibility to stress in PD patients. In mice, we have not observed any pathology in noradrenergic neurons of LC and only minimal α-syn aggregation in serotonergic neurons—which were reported to be affected in some PD patients [40], and these neuron populations are also critical structures in stress response. Importantly, most widely used antidepressants act by modulating serotonergic, noradrenergic, and/or dopaminergic systems. However, the functional significance of pathology in noradrenergic and serotonergic neurons in PD is relatively understudied. Also, while we have observed a modest loss of TH-positive cells in the SN and corresponding loss of dopaminergic innervation in the dorsal striatum, the VTA and the ventral striatum were not significantly affected—and these areas may be more linked with emotional regulation [41]. Nonetheless, a faster loss of cells in the SN than in the VTA is one of the features observed in PD patients, which validates that the dynamics of dopamine cell loss in our model are etiologically valid.

It is also possible that because the employed stress model was relatively modest, as evidenced by limited effects on behavior and immune parameters, it failed to interact with the induced α-syn-related pathology. On the other hand, an increase in susceptibility to stress should be best distinguishable when the stressor is modest, since stronger/longer stressor effects could be masked by ceiling effects. Stressor severity is somewhat difficult to control experimentally, as responsiveness to different kinds of stress procedures might be affected by the animal’s age, strain, sex, and other factors [42, 43]. Most stress protocols are adopted and tested on relatively young animals, while the progressive nature of PFF-induced pathology necessitated subjecting animals to stress after several months of pathology development—thus in more mature animals than usually utilized—which could explain relatively modest stress effects. On the other hand, it has been argued that even much older animals should be employed in α-syn aggregation models in vivo to better recapitulate PD etiology [44]. Furthermore, time of pathology progression from PFF injections could be further extended. However, in such cases, the experimenter have to take into account additional technical complications [44] apart from the poor characterization of stress models. Moreover, pathology in PD patients might start years, or even decades before diagnosis [1], this further complicates attempts to correlate the progression stage of PD with animal models. In our experiment, we decided to use time points when pathology in limbic structures is well pronounced. Nonetheless, exploring the importance of animal age and time of pathology development on non-motor symptoms and susceptibility to stress will be an important task for future studies. To avoid additional complications linked with the reliability of stress procedures in aged animals utilization of simplified, yet more controllable procedures—like chronic injections of corticosterone—might be good alternatives in future studies.

Also, timing of the stress might be important. We decided to subject the animals to chronic stress at the time point when α-syn aggregation is widespread in the brain, but motor symptoms are not yet prominent, thus mimicking to some extent pre-symptomatic PD when the depression starts to occur in patients. However, it is possible that stress applied at different time points, either earlier when Lewy pathology starts to develop or even later when loss of DA cells is more prominent, could elicit depressive symptoms in α-syn PFF-injected animals. Nonetheless, correlating the stage of pathology in animal α-syn PFF model to PD stages is difficult, as there are some conflicting reports on pathology staging in humans, while α-syn PFF does not completely recapitulate the spreading pattern of Lewy pathology in patients [45, 46]. It would be very interesting to test if Lewy pathology and cell loss in noradrenergic and serotonergic systems might affect the development of depressive symptoms. Lewy pathology in those regions was almost absent in our model, while it is reported at least in some subpopulations of PD patients [40]. This would require establishing of a novel model, possibly combining multiple α-syn PFF sites [47–49] or α-syn PFF injected into transgenic animals [50–52] to recapitulate pathology in a feasible timeframe.

We have not observed any effects of the chronic stress on the amount of α-syn aggregates in the SN, the frontal cortex, and the amygdala in α-syn PFF-injected animals. However, it should be noted that our experiment schedule was designed to explore the effects of already-developed pathology on the stress response, rather than the opposite. A paradigm, when stress or stress hormones are applied at the early stage after α-syn PFF injections, would be better suited to test the effect of stress on the formation and spreading of α-syn aggregates. We have assessed sections only from α-syn PFF-injected animals, but not from control or stressed PBS-injected animals. However, it is well established both in vitro and in vivo that Lewy-body-like α-syn aggregates form only after seeding with aggregated α-syn [24]. Even overexpression of α-syn leads only to the formation of diffuse aggregates, which do not resemble structures present in patient brains [39]. Nevertheless, it would be intriguing to investigate whether chronic stress could induce α-syn aggregation and the formation of Lewy Bodies. Such a study could be conducted in animals overexpressing α-syn to maximize the chances of detection. Finally, it should be noted that we have quantified the presence of pathological α-syn aggregation based on phosphor-Ser129- α-syn staining. This is well-established marker for Lewy bodies, which was also correlated by the other studies with the presence of other Lewy Body markers in animal PFF-induced α-syn aggregation [24, 53]. Nonetheless, other markers, and possibly advanced techniques like Correlative Light and Electron Microscopy would be required to draw possible conclusions if stress could have effects on the maturation process of aggregates or their composition [53, 54].

Finally, there are several further questions regarding α-syn that might also be relevant in the context of stress susceptibility and depression in Parkinson’s disease. The still-elusive physiological role of α-syn is one such major question. Apart from the putative toxic effects of misfolded alpha-synuclein, its aggregation might also deplete the correctly formed protein pool. Snca-null animals have so far shown only very minor abnormalities [54] but have not been investigated in the context of chronic stress. Such studies might become even more important as lowering endogenous α-syn levels is being pursued as a potential therapeutic intervention in Parkinson’s disease [56]. Furthermore, the role of different posttranslational modifications of α-syn in the aggregation process, conformation, and toxicity of the aggregated form is currently highly debated and intensively investigated [53]. The role of these modifications and different forms of α-syn in the context of non-motor symptoms of PD, like depression, will also need to be investigated in the future.

In summary, our data suggest that prominent α-syn aggregation in the amygdala and prefrontal cortex together with modest loss of dopamine neurons in the SNpc is insufficient to cause increased susceptibility to chronic stress. While early, perhaps compensatory changes in behavior are already observed. However, as discussed above, multiple considerations should be taken for interpreting the results in the context of depression in Parkinson’s disease. This is largely due to insufficient data regarding links between pathology in PD patient’s brains and depressive symptoms, as well as the scarcity of investigations in animal models. Furthermore, limitations inherent to modeling decades-long progression of pathology in much shorter lived animal models add to the complexity of the problem. Future studies should consider testing different time frames of the experiment with both longer progression after injection of α-syn-PFFs, and stress at different time points, as well as the use of aged animals. Such studies would be best made with models where α-syn-PFFs injections are optimized to observe pathology also in noradrenergic and serotonergic neurons, perhaps when peripheral α-syn-PFFs injection models become more reliable. Furthermore, independently of the interaction with stress, the paradoxical effect of increased locomotor activity should be investigated on a functional level as it might point to compensatory changes in dopamine release, uptake, or receptor density in the early stages of α-syn pathology, which could have implications for pre-symptomatic PD. Overall, we are clearly at the beginning of investigations into the role of α-syn aggregation in the increased prevalence of depression in PD.

### Supplementary Information

Below is the link to the electronic supplementary material.

Supplementary file1 (DOCX 10069 KB)

Supplementary file2 (PDF 7478 KB)

## Data Availability

The datasets generated during and/or analyzed during the current study are available from the corresponding author upon reasonable request.

## Abbreviations

CSD: Chronic social defeat
DA: Dopaminergic
DLB: Dementia with Lewy bodies
IL: Infralimbic
LBs: Lewy bodies
LC: Locus coeruleus
LD-box: Light–dark box
LNs: Lewy neurites
pc-SN; SNpc; SN: (Pars compacta) Substantia Nigra
PD: Parkinson’s disease
PFFs: Preformed fibrils
PL: Prelimbic
RN: Raphe nuclei
SI: Social interaction
TH +: Tyrosine hydroxylase positive
TST: Suspension test
VTA: Ventral tegmental area
α-syn: α-synuclein

## References

[1] Kalia LV, Lang AE (2015). Parkinson’s disease. Lancet.

[2] Balestrino R, Schapira AHV (2020). Parkinson disease. Eur J Neurol.

[3] Braak H, Braak E, Yilmazer D, de Vos RAI, Jansen ENH, Bohl J (1994). Amygdala pathology in Parkinson’s disease. Acta Neuropathol (Berl).

[4] Surmeier DJ, Obeso JA, Halliday GM (2017). Selective neuronal vulnerability in Parkinson disease. Nat Rev Neurosci.

[5] Shulman LM, Taback RL, Rabinstein AA, Weiner WJ (2002). Non-recognition of depression and other non-motor symptoms in Parkinson’s disease. Parkinsonism Relat Disord.

[6] Churchyard A, Lees AJ (1997). The relationship between dementia and direct involvement of the hippocampus and amygdala in Parkinson’s disease. Neurology.

[7] Mattila PM, Rinne JO, Helenius H, Dickson DW, Röyttä M (2000). Alpha-synuclein-immunoreactive cortical Lewy bodies are associated with cognitive impairment in Parkinson’s disease. Acta Neuropathol (Berl).

[8] Schmidt ML, Martin JA, Lee VM, Trojanowski JQ (1996). Convergence of Lewy bodies and neurofibrillary tangles in amygdala neurons of Alzheimer’s disease and Lewy body disorders. Acta Neuropathol (Berl).

[9] Aarsland D, Påhlhagen S, Ballard CG, Ehrt U, Svenningsson P (2012). Depression in Parkinson disease—epidemiology, mechanisms and management. Nat Rev Neurol.

[10] Akhmadeeva GN, Magzhanov RV, Tayupova GN, Baitimerov AR, Khidiyatova IM (2018). Depression and anxiety in Parkinson’s disease. Neurosci Behav Physiol.

[11] Alonso A, Rodríguez LAG, Logroscino G, Hernán MA (2009). Use of antidepressants and the risk of Parkinson’s disease: a prospective study. J Neurol Neurosurg Psychiatry.

[12] Chiu P-Y, Wang C-W, Tsai C-T, Li S-H, Lin C-L, Lai T-J (2017). Depression in dementia with Lewy bodies: a comparison with Alzheimer’s disease. PLoS ONE.

[13] Chmielarz P, Saarma M (2020). Neurotrophic factors for disease-modifying treatments of Parkinson’s disease: gaps between basic science and clinical studies. Pharmacol Rep PR.

[14] Lee S, Jeong J, Kwak Y, Park SK (2010). Depression research: where are we now?. Mol Brain.

[15] Surdhar I, Gee M, Bouchard T, Coupland N, Malykhin N, Camicioli R (2012). Intact limbic-prefrontal connections and reduced amygdala volumes in Parkinson’s disease with mild depressive symptoms. Parkinsonism Relat Disord.

[16] Frisina PG, Haroutunian V, Libow LS (2009). The neuropathological basis for depression in Parkinson’s disease. Parkinsonism Relat Disord.

[17] McShane RH, Nagy Z, Esiri MM, King E, Joachim C, Sullivan N (2001). Anosmia in dementia is associated with Lewy bodies rather than Alzheimer’s pathology. J Neurol Neurosurg Psychiatry.

[18] Borghammer P (2021). The α-synuclein origin and connectome model (SOC Model) of Parkinson’s disease: explaining motor asymmetry, non-motor phenotypes, and cognitive decline. J Park Dis.

[19] Volpicelli-Daley LA, Luk KC, Lee VM-Y (2014). Addition of exogenous α-synuclein preformed fibrils to primary neuronal cultures to seed recruitment of endogenous α-synuclein to Lewy body and Lewy neurite-like aggregates. Nat Protoc.

[20] Polinski NK (2021). A summary of phenotypes observed in the in vivo rodent alpha-synuclein preformed fibril model. J Park Dis.

[21] Merola A, Romagnolo A, Dwivedi AK, Padovani A, Berg D, Garcia-Ruiz PJ (2020). Benign versus malignant Parkinson disease: the unexpected silver lining of motor complications. J Neurol.

[22] Ross RA, Foster SL, Ionescu DF (2017). The role of chronic stress in anxious depression. Chronic Stress Thousand Oaks Calif.

[23] Tafet GE, Nemeroff CB (2016). The links between stress and depression: psychoneuroendocrinological, genetic, and environmental interactions. J Neuropsychiatry Clin Neurosci.

[24] Luk KC, Kehm V, Carroll J, Zhang B, O’Brien P, Trojanowski JQ (2012). Pathological α-synuclein transmission initiates Parkinson-like neurodegeneration in nontransgenic mice. Science.

[25] Polinski NK, Volpicelli-Daley LA, Sortwell CE, Luk KC, Cremades N, Gottler LM (2018). Best practices for generating and using alpha-synuclein pre-formed fibrils to model Parkinson’s disease in rodents. J Park Dis.

[26] Er S, Hlushchuk I, Airavaara M, Chmielarz P, Domanskyi A (2020). Studying pre-formed fibril induced α-synuclein accumulation in primary embryonic mouse midbrain dopamine neurons. J Vis Exp JoVE.

[27] Chmielarz P, Kuśmierczyk J, Parlato R, Schütz G, Nalepa I, Kreiner G (2013). Inactivation of glucocorticoid receptor in noradrenergic system influences anxiety- and depressive-like behavior in mice. PLoS ONE.

[28] Schindelin J, Arganda-Carreras I, Frise E, Kaynig V, Longair M, Pietzsch T (2012). Fiji: an open-source platform for biological-image analysis. Nat Methods.

[29] Arganda-Carreras I, Kaynig V, Rueden C, Eliceiri KW, Schindelin J, Cardona A (2017). Trainable Weka Segmentation: a machine learning tool for microscopy pixel classification. Bioinformatics.

[30] Jankowska-Kieltyka M, Roman A, Mikrut M, Kowalska M, van Eldik R, Nalepa I (2021). Metabolic response of RAW 264.7 macrophages to exposure to crude particulate matter and a reduced content of organic matter. Toxics.

[31] Choi C, Sohn YH, Lee JH, Kim J-S (2000). The effect of long-term levodopa therapy on depression level in de novo patients with Parkinson’s disease. J Neurol Sci.

[32] Golden JP, DeMaro JA, Knoten A, Hoshi M, Pehek E, Johnson EM (2013). Dopamine-dependent compensation maintains motor behavior in mice with developmental ablation of dopaminergic neurons. J Neurosci.

[33] Perez XA, Parameswaran N, Huang LZ, O’Leary KT, Quik M (2008). Pre-synaptic dopaminergic compensation after moderate nigrostriatal damage in non-human primates. J Neurochem.

[34] Bezard E, Jaber M, Gonon F, Boireau A, Bloch B, Gross CE (2000). Adaptive changes in the nigrostriatal pathway in response to increased 1-methyl-4-phenyl-1,2,3,6-tetrahydropyridine-induced neurodegeneration in the mouse. Eur J Neurosci.

[35] Stoyka LE, Arrant AE, Thrasher DR, Russell DL, Freire J, Mahoney CL (2020). Behavioral defects associated with amygdala and cortical dysfunction in mice with seeded α-synuclein inclusions. Neurobiol Dis.

[36] Chesselet M-F, Richter F, Zhu C, Magen I, Watson MB, Subramaniam SR (2012). A progressive mouse model of Parkinson’s disease: the Thy1-aSyn (“Line 61”) mice. Neurother J Am Soc Exp Neurother.

[37] Magen I, Torres ER, Dinh D, Chung A, Masliah E, Chesselet M-F (2015). Social cognition impairments in mice overexpressing alpha-synuclein under the Thy1 promoter, a model of pre-manifest Parkinson’s disease. J Park Dis.

[38] Magen I, Fleming SM, Zhu C, Garcia EC, Cardiff KM, Dinh D (2012). Cognitive deficits in a mouse model of pre-manifest Parkinson’s disease. Eur J Neurosci.

[39] Fares MB, Jagannath S, Lashuel HA (2021). Reverse engineering Lewy bodies: how far have we come and how far can we go?. Nat Rev Neurosci.

[40] Buddhala C, Loftin SK, Kuley BM, Cairns NJ, Campbell MC, Perlmutter JS (2015). Dopaminergic, serotonergic, and noradrenergic deficits in Parkinson disease. Ann Clin Transl Neurol.

[41] MacDonald PA, Monchi O (2011). Differential effects of dopaminergic therapies on dorsal and ventral striatum in Parkinson’s disease: implications for cognitive function. Park Dis.

[42] Kreiner G, Chmielarz P, Roman A, Nalepa I (2013). Gender differences in genetic mouse models evaluated for depressive-like and antidepressant behavior. Pharmacol Rep PR.

[43] Chmielarz P, Kreiner G, Kuśmierczyk J, Kowalska M, Roman A, Tota K (2016). Depressive-like immobility behavior and genotype × stress interactions in male mice of selected strains. Stress Amst Neth.

[44] Klæstrup IH, Just MK, Holm KL, Alstrup AKO, Romero-Ramos M, Borghammer P (2022). Impact of aging on animal models of Parkinson’s disease. Front Aging Neurosci.

[45] Taguchi T, Ikuno M, Yamakado H, Takahashi R (2020). Animal model for prodromal Parkinson’s disease. Int J Mol Sci.

[46] Zhang TD, Kolbe SC, Beauchamp LC, Woodbridge EK, Finkelstein DI, Burrows EL (2022). How well do rodent models of Parkinson’s disease recapitulate early non-motor phenotypes? A systematic review. Biomedicines.

[47] Challis C, Hori A, Sampson TR, Yoo BB, Challis RC, Hamilton AM (2020). Gut-seeded α-synuclein fibrils promote gut dysfunction and brain pathology specifically in aged mice. Nat Neurosci.

[48] Wang X-J, Ma M-M, Zhou L-B, Jiang X-Y, Hao M-M, Teng RKF (2020). Autonomic ganglionic injection of α-synuclein fibrils as a model of pure autonomic failure α-synucleinopathy. Nat Commun.

[49] Kim S, Kwon S-H, Kam T-I, Panicker N, Karuppagounder SS, Lee S (2019). Transneuronal propagation of pathologic α-synuclein from the gut to the brain models Parkinson’s disease. Neuron.

[50] Butkovich LM, Houser MC, Chalermpalanupap T, Porter-Stransky KA, Iannitelli AF, Boles JS (2020). Transgenic mice expressing human α-synuclein in noradrenergic neurons develop locus ceruleus pathology and nonmotor features of Parkinson’s disease. J Neurosci.

[51] Deusser J, Schmidt S, Ettle B, Plötz S, Huber S, Müller CP (2015). Serotonergic dysfunction in the A53T alpha-synuclein mouse model of Parkinson’s disease. J Neurochem.

[52] Barut J, Rafa-Zabłocka K, Jurga AM, Bagińska M, Nalepa I, Parlato R (2022). Genetic lesions of the noradrenergic system trigger induction of oxidative stress and inflammation in the ventral midbrain. Neurochem Int.

[53] Mahul-Mellier A-L, Burtscher J, Maharjan N, Weerens L, Croisier M, Kuttler F (2020). The process of Lewy body formation, rather than simply α-synuclein fibrillization, is one of the major drivers of neurodegeneration. Proc Natl Acad Sci.

[54] Lashuel HA (2021). Rethinking protein aggregation and drug discovery in neurodegenerative diseases: why we need to embrace complexity?. Curr Opin Chem Biol.

