# Supplementary material for: Investigating the potential effects of α-synuclein aggregation on susceptibility to chronic stress in a mouse Parkinson’s disease model
Source: Pharmacol Rep. 2023 Sep 19;75(6):1474–87. doi: 10.1007/s43440-023-00530-z (PMC10661792; doi:10.1007/s43440-023-00530-z)
Supplement: Supplementary file 1 — Supplementary file1 (DOCX 10069 KB) [file 43440_2023_530_MOESM1_ESM.docx]

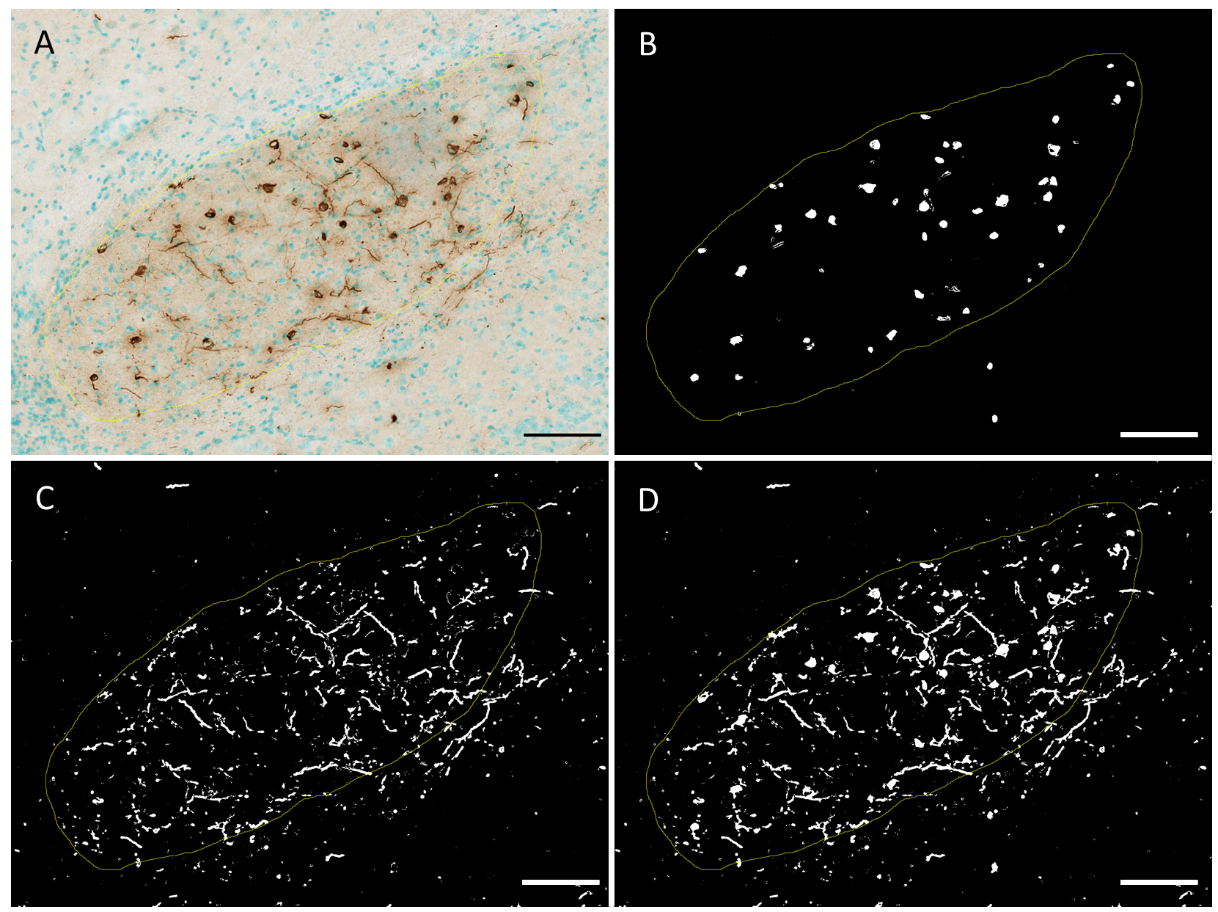


**Figure S1.** Example quantification procedure of Lewy pathology in the amygdala region (A). Masks encompassing Lewy Bodies (B) and Lewy Neurites (C), or the total area covered by αsyn aggregates (D) were obtained utilizing Trainable Weka Segmentation 2D (TWS) plugin in FIJI. Scale bars = 100 µm


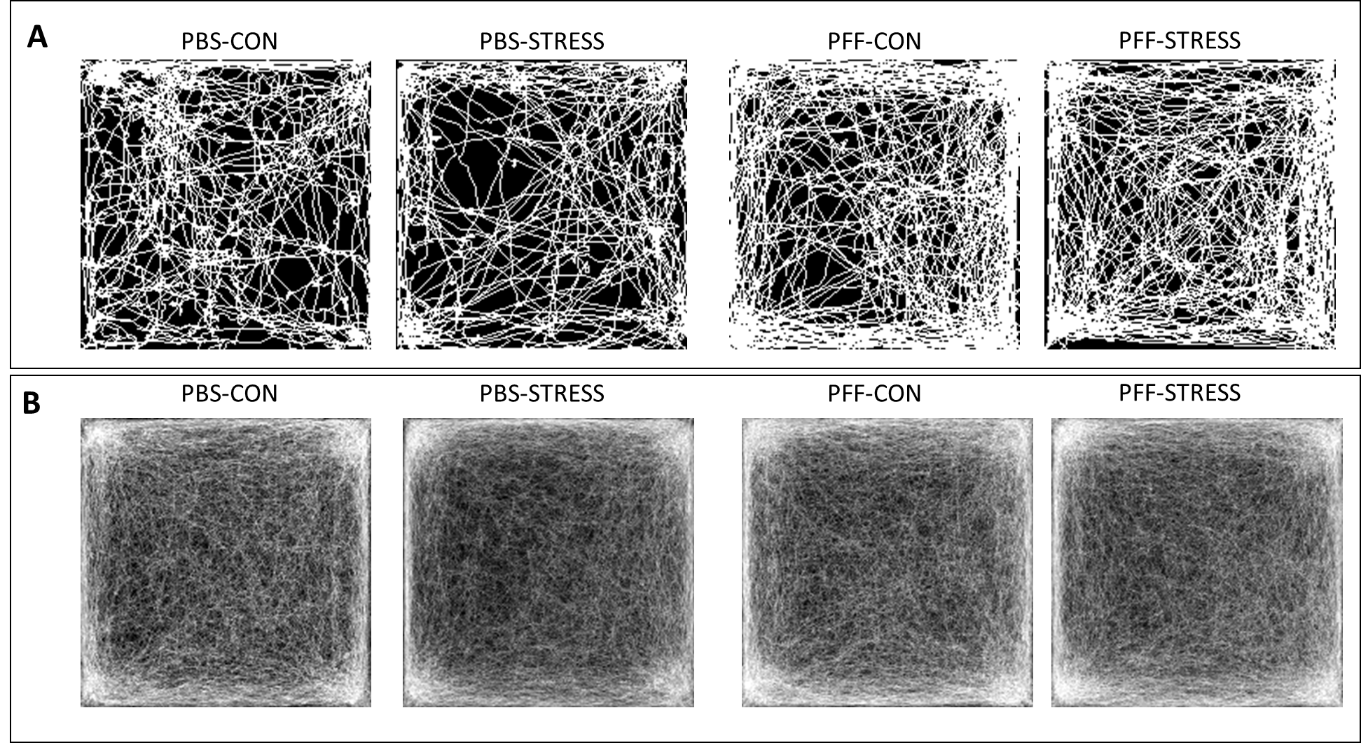


**Figure S2.** Tracks acquired from open field test. A) example tracks of mice in open field from each experimental group B) Averaged tracks of all animals in group


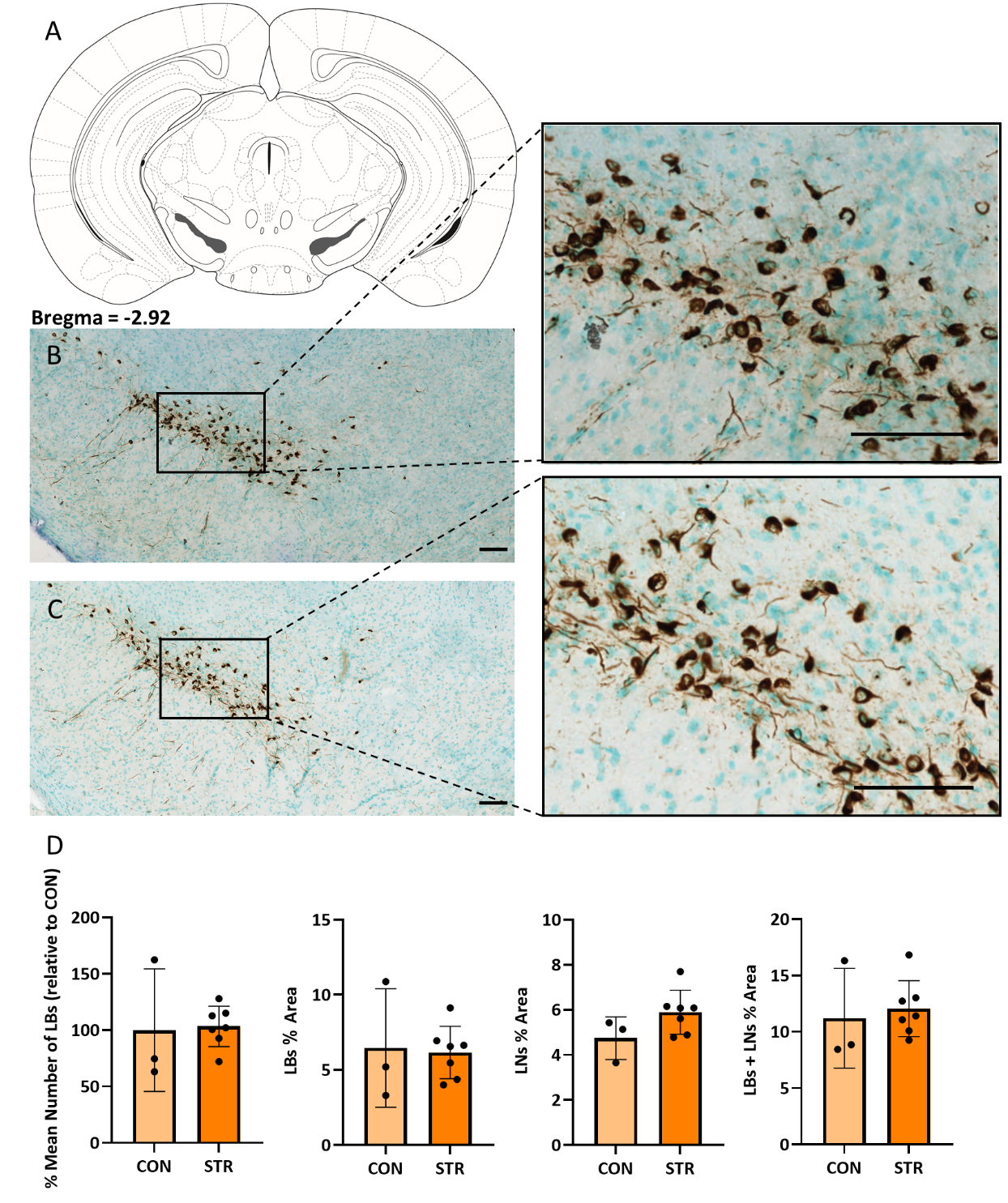


**Figure S3** The comparison of LB and LN (brown) inclusions in the pc-SN (A, marked in grey) after PFF injections in non-stressed (CON) (B, D) and stressed (STR) (C, D) groups in the brain sections stained against pS129α-syn (brown) and counterstained with methyl green (blue-green). N=3-7. Data are mean ±SD, Scale bars = 100 µm.


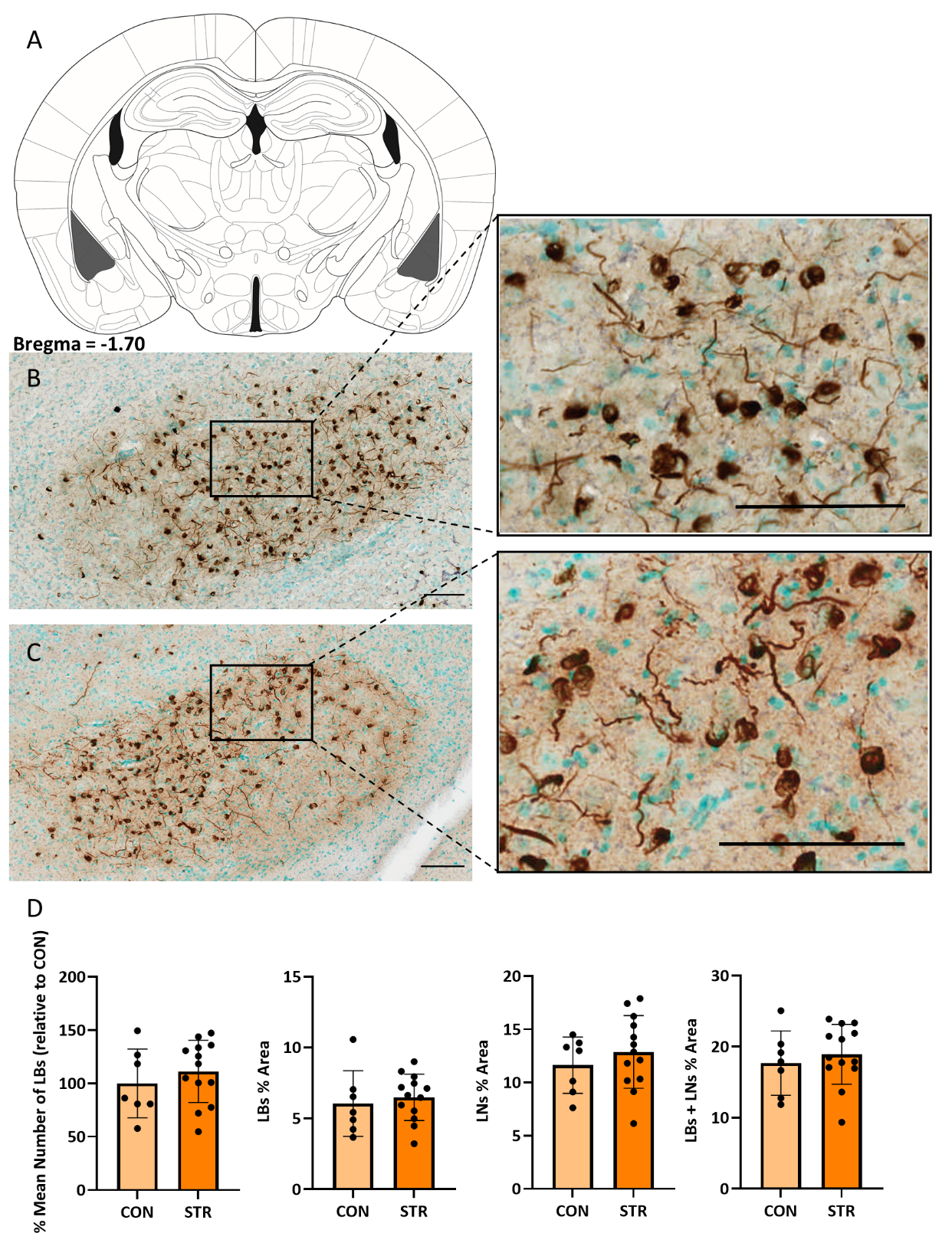


**Figure S4** The comparison of LB and LN (brown) inclusions in the amygdala (A, marked in grey) after PFF injections between stressed (STR) (C, D) and non-stressed (CON) (B, D) groups in the brain sections stained against pS129α-syn (brown) and counterstained with methyl green (blue-green). No significant changes were observed in the inclusion numbers nor the area covered by LBs and LNs. N=7-13. Data are mean ±SD, Scale bars = 100 µm


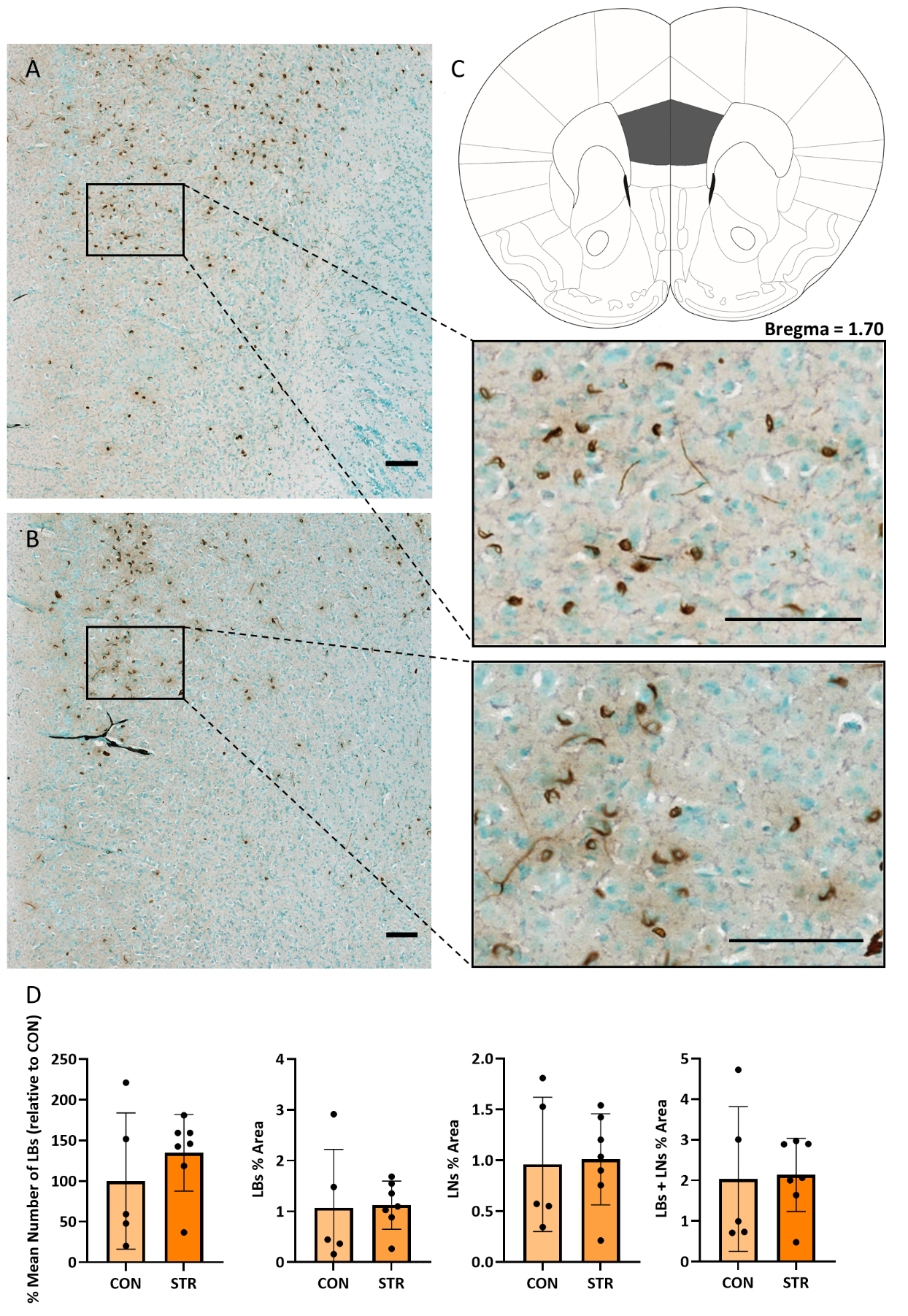


**Figure S5** The comparison of LB and LN (brown) inclusions in the PL and IL cortex (C, marked in grey) after PFF injections between non-stressed (CON) (A, D) and stressed (STR) (B, D) groups in the brain sections stained against pS129α-syn (brown) and counterstained with methyl green (blue-green). N=5-7/ Data are mean ±SD, Scale bars = 100 µm.


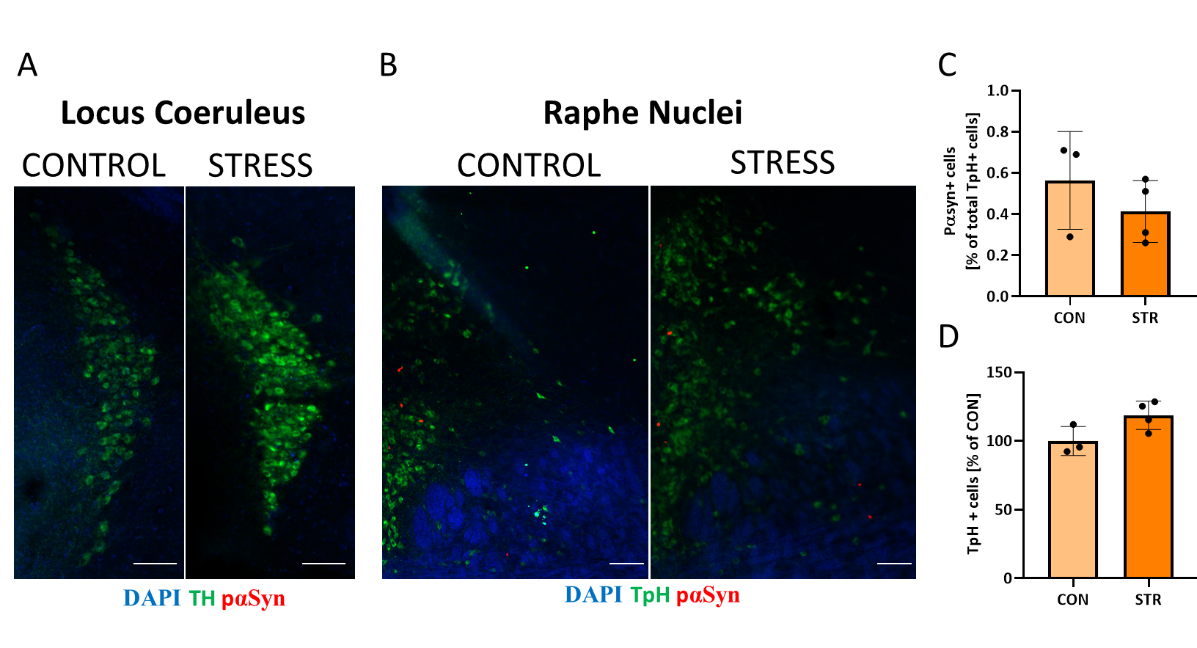


**Figure S6** Visualisation of α-syn aggregation, or lack thereof, in noradrenergic neurons of Locus Coeruleus (LC) and serotonergic neurons of Raphe Nuclei (RN) of control and stressed, PFF injected animals. (A) Representative images of tyrosine hydroxylase (TH, green) and or pS129-αsyn (pαSyn , red) in LC. (B) Representative images of tryptophan hydroxylase (TpH, green) and pS129-αsyn (pαSyn , red) in RN. (C) Percentage of TpH-positive cells containing αsyn aggregates. (D) total count of TpH positive cells in the RN. N=3-4. Data are mean ±SD, Scale bars = 100µm
