# Supplementary material for: Investigating the potential effects of α-synuclein aggregation on susceptibility to chronic stress in a mouse Parkinson’s disease model
Source: Pharmacol Rep. 2023 Sep 19;75(6):1474–87. doi: 10.1007/s43440-023-00530-z (PMC10661792; doi:10.1007/s43440-023-00530-z)
Supplement: Supplementary file 2 — Supplementary file2 (PDF 7478 KB) [file 43440_2023_530_MOESM2_ESM.pdf]

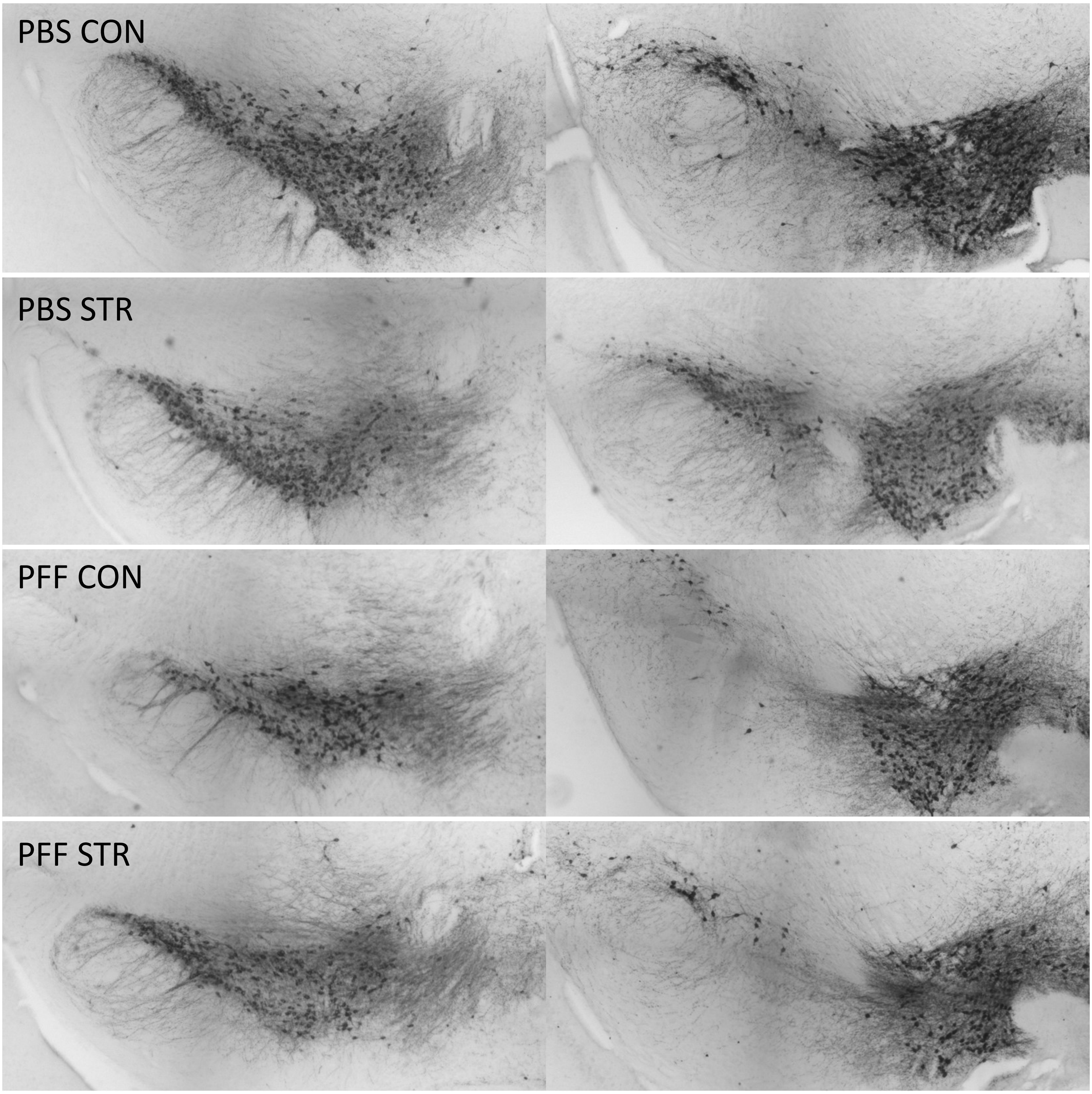

Figure S7. Additional exampel images of Thyrosine Hydroxilase stained sections from different areas of Substantia Nigra. PBS CON – PBS injected non-stressed animals; PBS STR – PBS injected stressed animals; PFF CON – PFF injected non-stressed animals; PFF STR – PFF injected stressed animals.

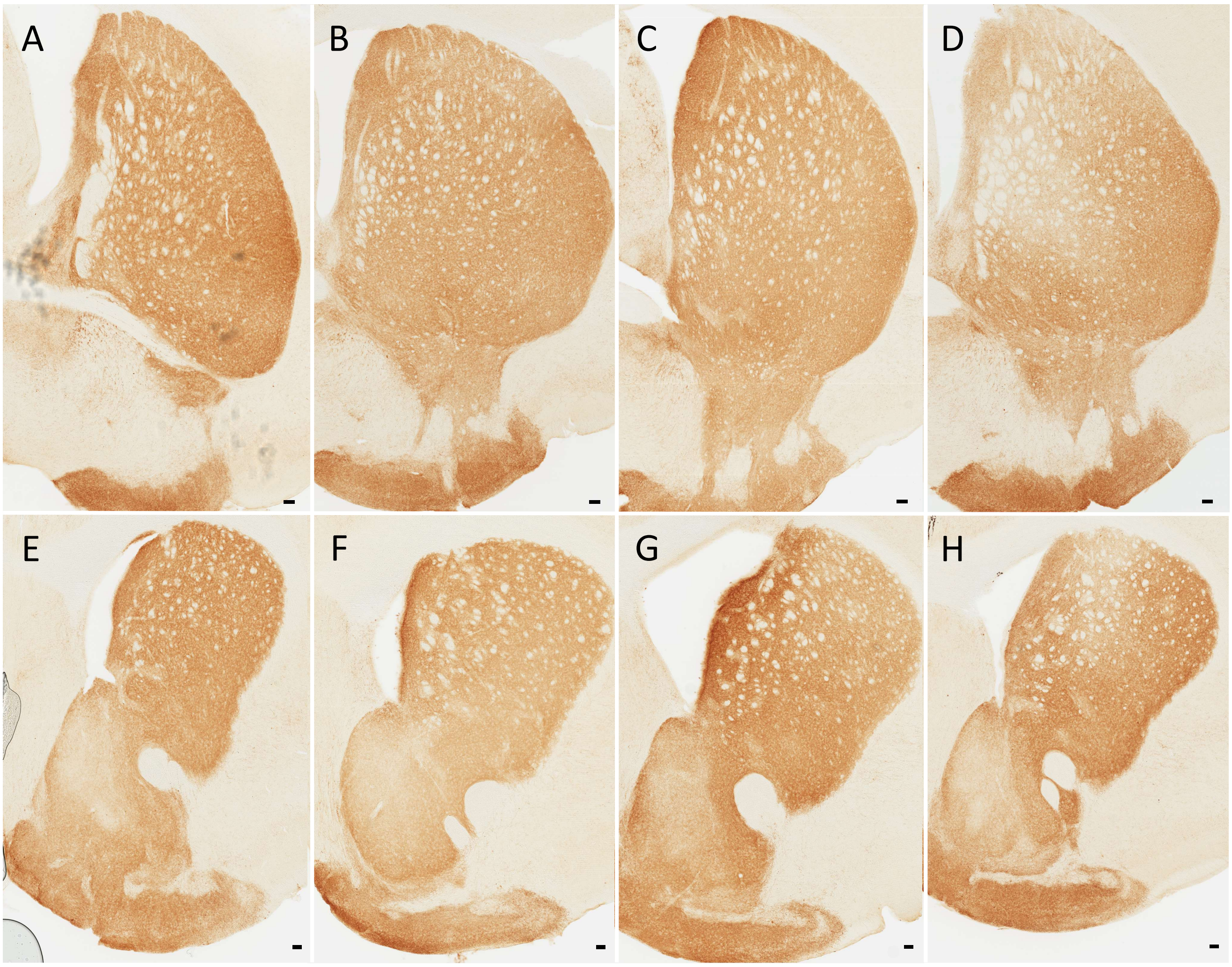

Figure S8. Additional representative images of immunohistochemical evaluation of Tyrosine Hydroxylase positive (TH+) fibers (brown) in the striatum of unstressed control (CON) or chronically stressed (STR) animals injected bilaterally either with PBS or αsyn-PFFs (PFF). A-D) bregma = 0.02; E-H) bregma = 1.54 A, E) CON/PBS animals B, D) STR/PBS animals, C, G) CON/PFF animals and D, H) STR/PFF animals. Scale bars = 100μm.

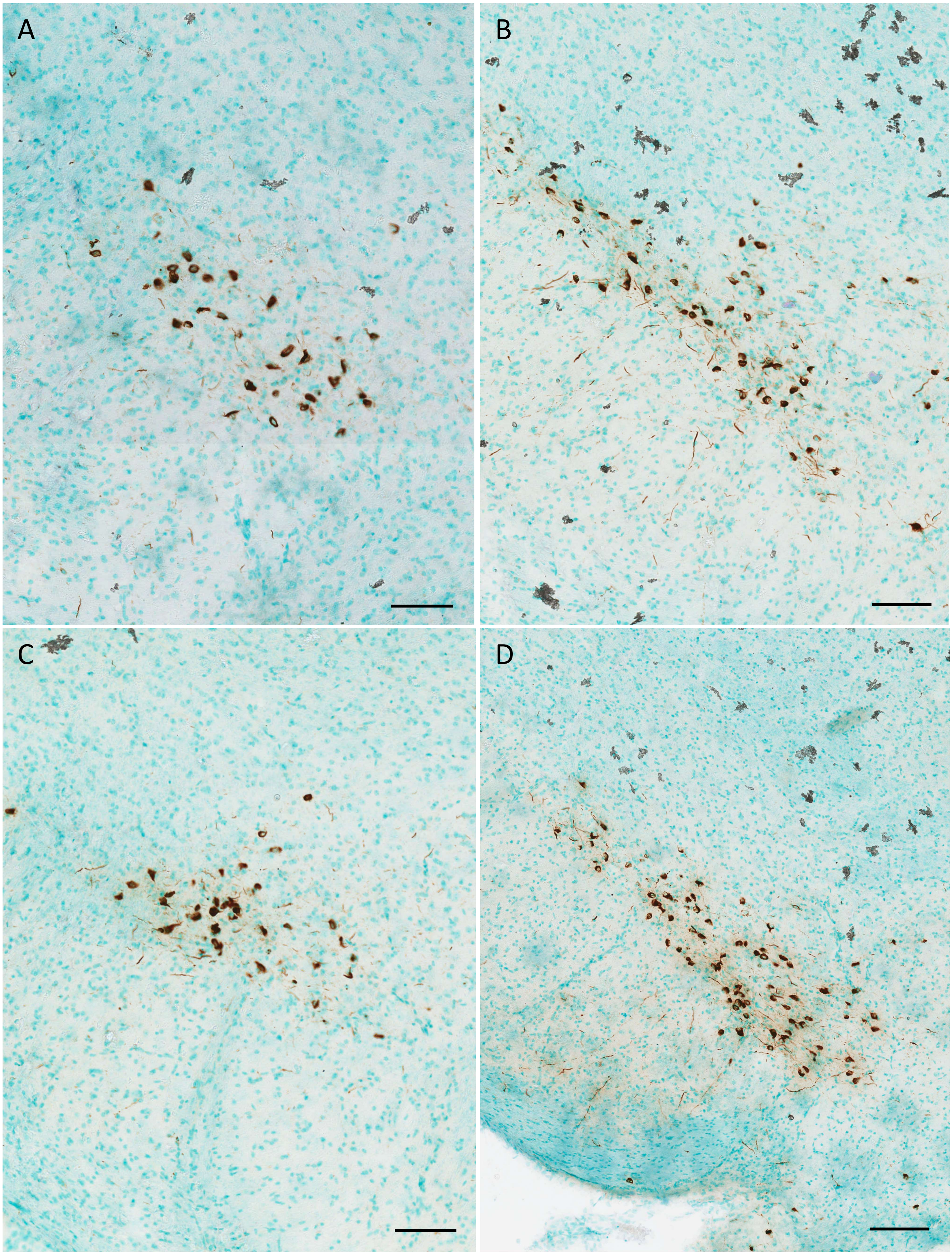

Figure S9. Additional representative images of the LB and LN (brown) inclusions in the pc-SN between non-stressed PFF injected (CON) (A, B) and stressed PFF injected (STR) (C, D) groups in the brain sections (bregma =-2.70 (A, C) and -2.80 (B, D)) stained against pS129α-syn (brown) and counterstained with methyl green (blue-green). Scale bars = 100 μm.

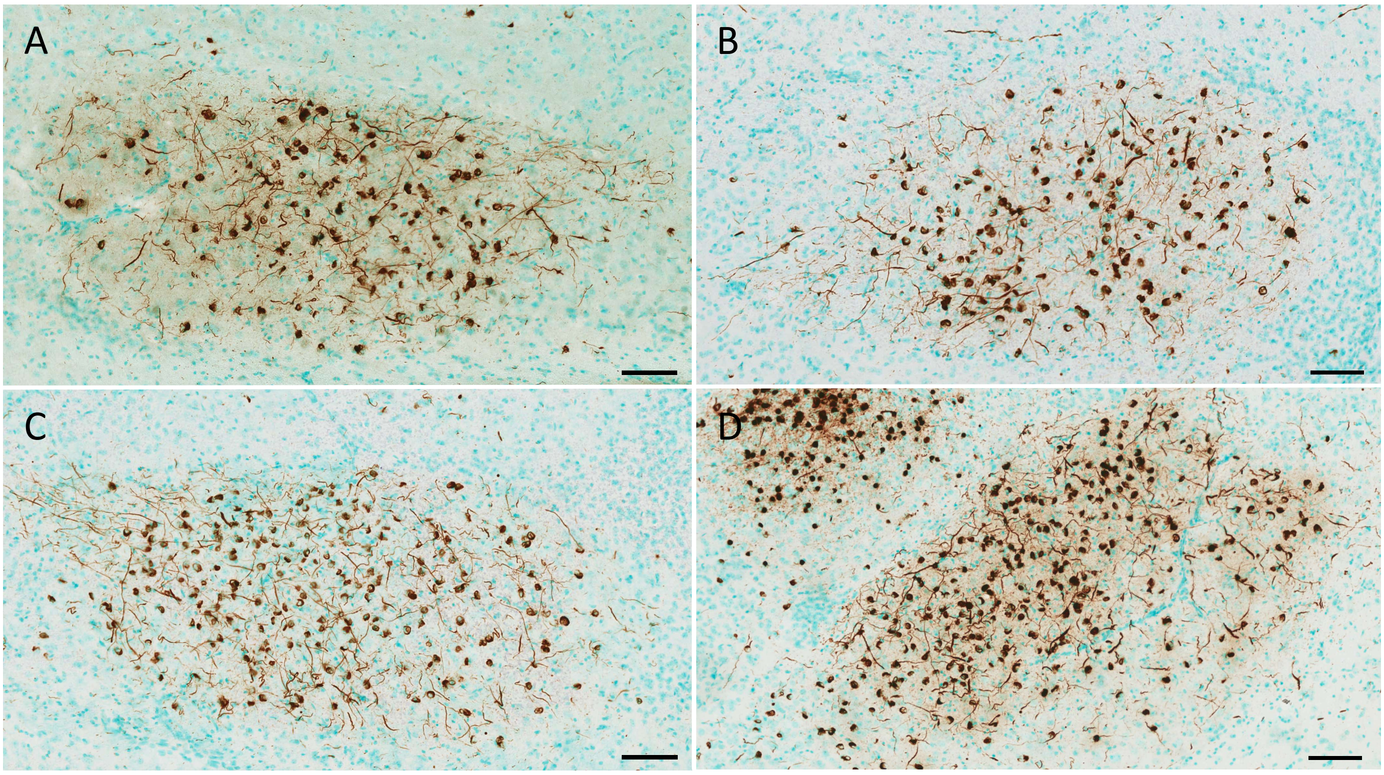

Figure S10. Additional representative images of the LB and LN (brown) inclusions in the amygdala between non-stressed (CON) (B, D) and stressed (STR) (C, D) groups in the brain sections (bregma = -0.94 (A, C) and -2.06 (B, D)) stained against pS129α-syn (brown) and counterstained with methyl green (blue-green). Scale bars = 100 μm.

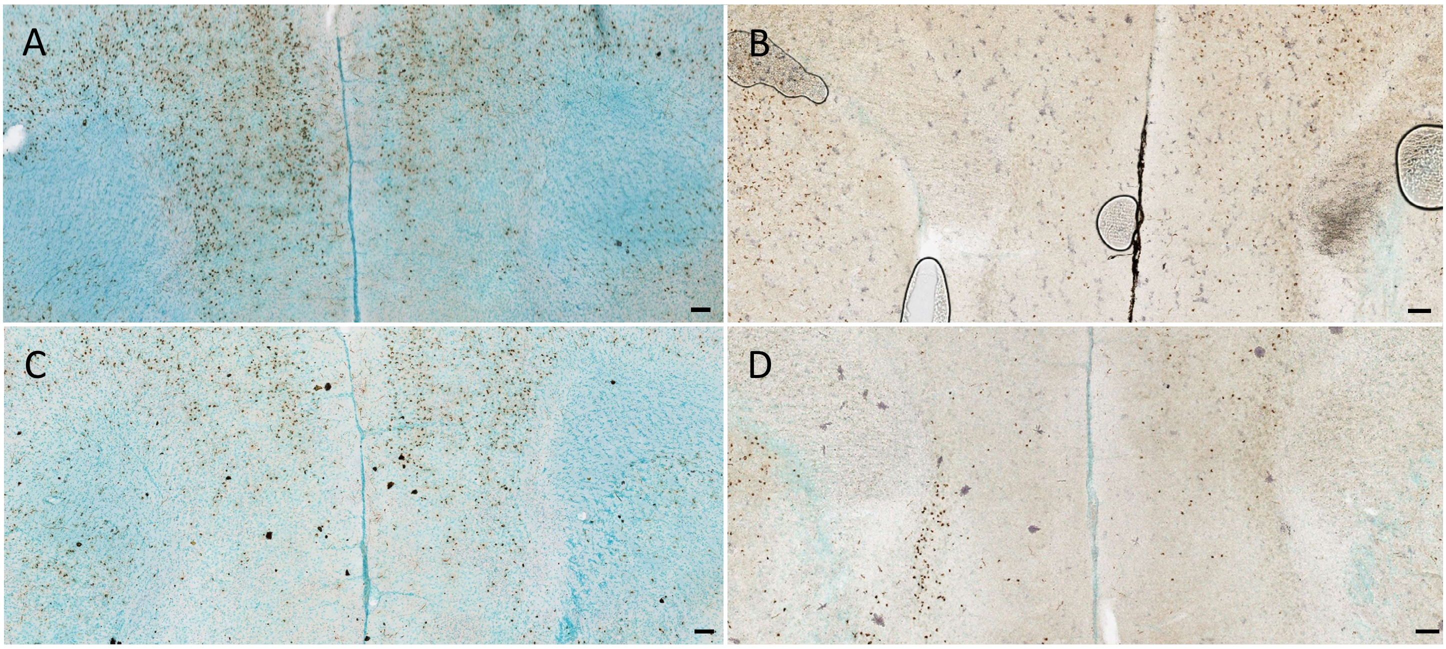

Figure S11. Additional representative images of the LB and LN (brown) inclusions in the PL and IL cortex between non-stressed (CON) (A, B) and stressed (STR) (C, D) groups in the brain sections (bregma = 1.94 (A, C) and 1.70 (B, D)) stained against pS129α-syn (brown) and counterstained with methyl green (blue-green). Scale bars = 100 μm.

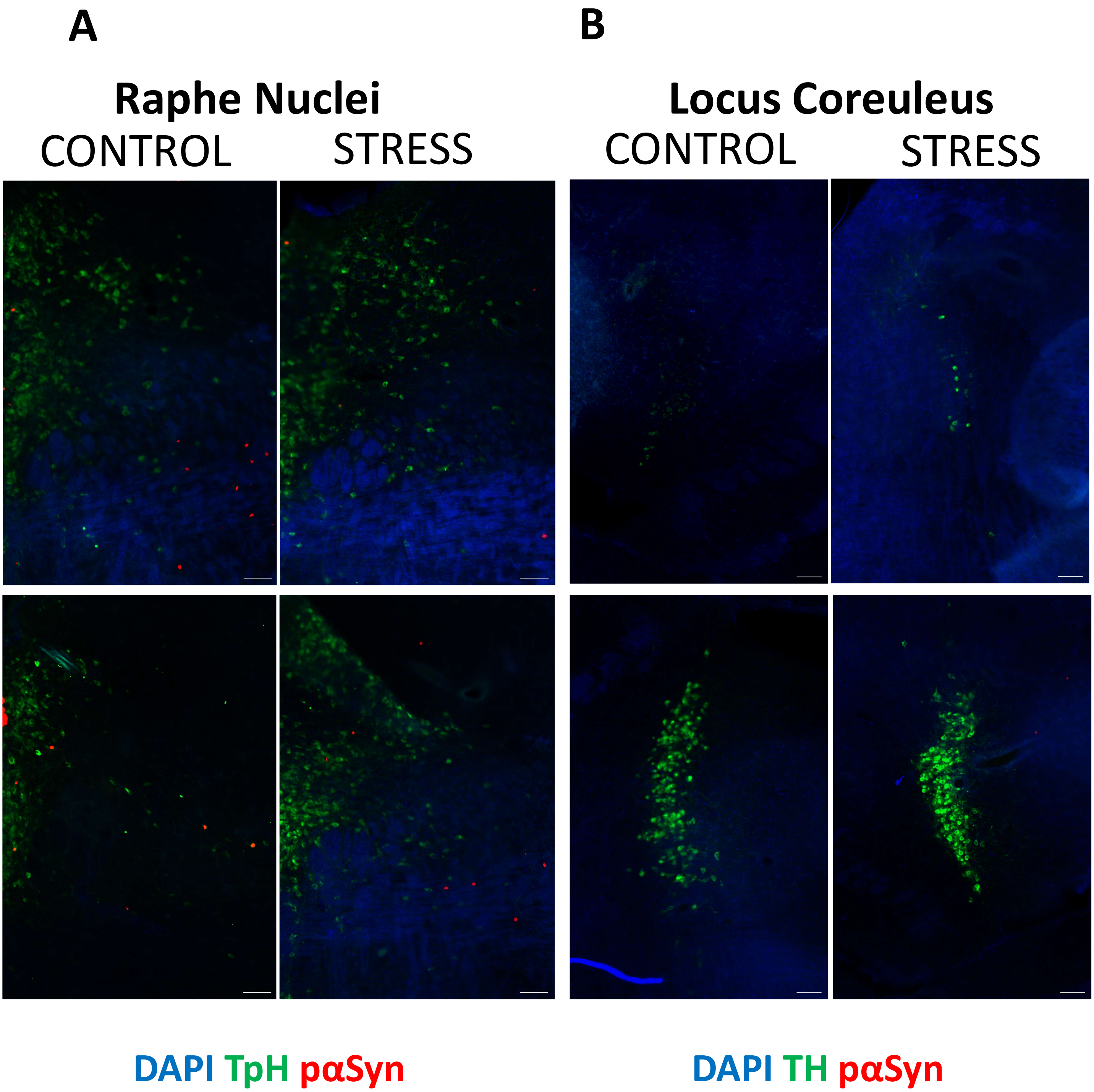

Figure S12. Additional representative images of α-syn aggregation, or lack thereof, in serotonergic neurons of Raphe Nuclei (RN) and noradrenergic neurons of Locus Coeruleus (LC) of control and stressed, PFF injected animals. (A) Representative images of tryptophan hydroxylase (TpH, green) and pS129-αsyn (pαSyn , red) in RN. (B) Representative images of tyrosine hydroxylase (TH, green) and or pS129-αsyn (pαSyn , red) in LC. Scale bars = 100µm
